# A Global Analysis of Tandem 3′UTRs in Eosinophilic Chronic Rhinosinusitis with Nasal Polyps

**DOI:** 10.1371/journal.pone.0048997

**Published:** 2012-11-19

**Authors:** Peng Tian, Yu Sun, Yuxin Li, Xiang Liu, Liang Wan, Jie Li, Yun Ma, Anlong Xu, Yonggui Fu, Hua Zou

**Affiliations:** 1 Department of Otorhinolaryngology-Head and Neck Surgery, Sun Yat-sen Memorial Hospital, Sun Yat-sen University, Guangzhou, P.R. China; 2 State Key Laboratory for Biocontrol, Guangdong Province Key Laboratory of Pharmaceutical Functional Genes, Department of Biochemistry, College of Life Sciences, Sun Yat-sen University, Higher Education Mega Center, Guangzhou, P.R. China; The John Curtin School of Medical Research, Australia

## Abstract

**Background:**

Alternative polyadenylation (APA) is emerging as a widespread mechanism of gene regulation. The usage of APA sites allows a single gene to encode multiple mRNA transcripts with different 3′-untranslated region (3′UTR) lengths. Many disease processes reflect the importance of the regulation of APA site switching. The objective of this study was to explore the profiling of tandem APA sites in nasal polyps compared with nasal uncinate process mucosa.

**Methods:**

Sequencing of APA sites (SAPAS) based on second-generation sequencing technology was undertaken to investigate the use of tandem APA sites and identify gene expression patterns in samples from the nasal polyps and nasal uncinate process mucosa of two patients with chronic rhinosinusitis with nasal polyps. The findings of the SAPAS analysis were validated via quantitative reverse-transcription polymerase chain reaction (qRT-PCR).

**Results:**

First, the results showed a switching of 3′UTR lengths in nasal polyps compared with nasal uncinate process mucosa. From the two patients, 105 genes that were detected in both patients in the nasal polyps were switched to distal poly(A) sites, and 90 such genes were switched to proximal poly(A) sites. Several Gene Ontology terms were enriched in the list of genes with switched APA sites, including transcription regulation, cell cycle, apoptosis, and metabolism. Second, we detected genes that showed differential expression with at least a 3-fold difference between nasal polyp tissue and nasal uncinate process mucosa. Between the two sample types, 627 genes exhibited differential expression. The qRT-PCR results confirmed our SAPAS results.

**Conclusion:**

APA site-switching events of 3′UTRs are prevalent in nasal polyp tissue, and the regulation of gene expression mediated by APA may play an important role in the formation and persistence of nasal polyps. Our results may provide new insights into the possible pathophysiologic processes involved in nasal polyps.

## Introduction

Chronic rhinosinusitis with nasal polyps (CRSwNP) is a common disease of the upper airway [1]. Nasal polyps, which are almost always present in conjunction with chronic rhinosinusitis (CRS), most often originate from the middle meatus and the ethmoid sinus region of the nasal cavity. Histologically, nasal polyps are characterized by inflammatory cell infiltration (eg, eosinophils, lymphocytes, and plasma cells), goblet cell hyperplasia, extracellular matrix protein accumulation, glandular hyperplasia, and edema [1]. The pathogenesis of this disease remains largely unknown. In recent years, many published studies have revealed that the development and persistence of nasal polyps are associated with numerous genes, the products of which determine various pathological processes, such as cytokine synthesis; immuno-pathogenesis; immune cell (e.g., lymphocyte, eosinophil, and neutrophil) development, activation, migration, and life span; adhesion molecule expression; and processes governing fibrosis and epithelial remodeling [2], [3], [4], [5]. With advances in microarray techniques, gene expression profiling of nasal polyp tissue has been performed, and novel genes related to nasal polyp formation have been identified. The large volume of published research and the complexity of the molecular interactions involved present a challenge to uncovering the mechanisms by which this network of gene expression is orchestrated.

The expression of gene products is regulated not only through changes in the rate of transcription but also by the stability and translational activity of mRNA transcripts. The 3′UTRs of mRNAs contain various cis-acting elements that influence mRNA metabolism via interaction with trans-acting factors, e.g., miRNA [6]. Over half of all human genes possess multiple alternative polyadenylation (APA) sites, which are poly (A) sites that generate multiple mRNA isoforms from a single gene [7]. The use of tandem APA sites located on the terminal exon often leads to tandem 3′UTRs with variable lengths. Tandem 3′UTRs play an important role in regulating the gene expression network because alternative mRNA isoforms that differ in their 3′UTRs can differ in their stability or translational activity [8]. Recent studies have shown that activated T lymphocytes [9] and cancer cells [10] are prone to using the shorter 3′UTR through APA and that shorter 3′UTRs are associated with cell proliferation [9]. Moreover, it was shown that APA might also be a mechanism by which certain proto-oncogenes are activated in cancer cells [10].

Although tandem APA-switching events have been found in activated immune cells and cancer, little is known about whether APA sites play an important role in nasal polyp tissue-regulated expression profiles compared with paired uncinate process tissue. In this study, the genome-wide tandem APA sites in nasal polyp tissue and the paired mucosa of the uncinate process derived from eosinophilic CRSwNP patients were examined using a novel strategy of sequencing APA sites (SAPAS) based on second-generation sequencing. We identified a large set of genes with 3′UTRs that varied in length between nasal polyp tissue from eosinophilic CRSwNP patients and control tissue. We also validated the results using quantitative RT-PCR in additional 10 patients.

## Results

### (1) Clinical manifestations

**Figure 1:**
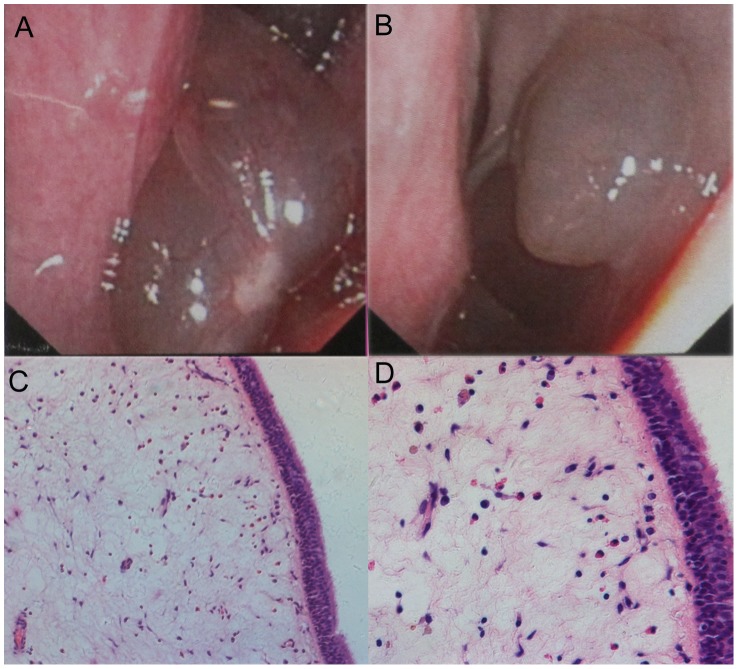
Histological analysis showing the types of nasal polyps in Chinese patients: eosinophilic nasal polyps. A). Nasal endoscopic findings; B). Nasal endoscopic findings; C). Histological appearance at 200×magnification; D). Histological appearance at 400×magnification.

Twelve patients who were diagnosed with chronic rhinosinusitis with nasal polyps (CRSwNPs) were selected for this study. These patients showed negative prick tests and an absence of allergies in their personal histories. They exhibited typically semitransparent nasal lesions that arose from the mucosa of the middle nasal meatus (Figure 1A and B). The clinical characteristics of all twelve patients are outlined in Table 1. Histologically, more than 100 eosinophils were visible at 200× magnification under light microscopy in every polyp sample [11], and clusters of eosinophils were observed (Figure 1C and D). The small sample size was chosen because this project was an exploratory study and initial analysis.

**Table 1:** The clinical characteristics of the patients with nasal polyps for SAPAS and q-PCR analysis.

Case	Age/Gender	Nasal obstruction	Nasal discharge	Loss of smell	Facial pain/pressure	Skin prick test	Asthma in history	Duration of disease	Polyp score (Davos)	CT score (Lund–Mackay)
#13*	46/M	moderate	slight	moderate	slight	-	-	10Y	5	13
#25*	46/M	moderate	slight	moderate	slight	-	-	20Y	4	9
#11§	40/F	slight	slight	slight	no symptom	-	-	10Y	6	8
#26§	52/M	severe	moderate	severe	slight	-	-	8Y	6	11
#37§	39/M	severe	moderate	slight	slight	-	-	12Y	4	14
#10§	35/F	slight	moderate	slight	slight	-	-	9Y	7	7
#46§	33/M	slight	slight	slight	moderate	-	-	12Y	5	8
#74§	45/F	severe	slight	slight	moderate	-	-	10Y	7	13
#58§	48/M	slight	moderate	severe	slight	-	-	8Y	3	10
#59§	50/F	severe	slight	slight	slight	-	-	10Y	7	8
#67§	46/M	moderate	slight	severe	moderate	-	-	20Y	6	9
#72§	43/M	moderate	moderate	slight	slight	-	-	5Y	5	9

* : Samples used for sequencing.

§ : Samples used for q-PCR. M: male; F: female. -: no atopic status, no asthma. Y: year. The atopic status was evaluated by skin prick tests to common inhalant allergens. The diagnosis of asthma was performed by a pneumologist. The CT score was obtained using the Lund-Mackay classification. The polyp size was scored according to the Davos classification.

### (2) Deep sequencing analysis of the 3′ends of mRNA

Using the SAPAS strategy [12], we profiled the APA sites of nasal polyp tissue and the adjacent nasal mucosa tissue. In total, 63.7 million raw reads with lengths of 75 bp were obtained using the Illumina sequencing platform (Table 2). Approximately 54.8 million reads (85.9%) harbored the modified anchor oligo d(T), approximately 38 million (58.9%) of which were uniquely mapped to the human nuclear genome (hg19). After filtering the reads with internal priming, 35.0 million reads (50.0%) could be used to directly infer transcript cleavage sites (Table 2).

**Table 2:** Summary statistics of the SAPAS data from Illumina GA IIx sequencing.

	Case 1		Case 2		Combined
	Nasal polyp	Control	Nasal polyp	Control	
Raw reads	19,145,727	15,364,528	20,392,147	8,837,913	63,740,315
Qualified reads	16,022,019	14,195,650	17,473,107	7,077,926	54,768,702 (85.9%)
Mapped to genome:	14,087,277	13,743,107	16,072,832	5,101,659	49,004,875 (76.9%)
Uniquely mapped to genome:	9,999,128	11,269,643	12,521,218	3,747,067	37,537,056 (58.9%)
Mapped to nuclear genome:	9,740,004	11,111,528	12,378,792	3,688,180	36,918,504 (57.9%)
After IP filter*:	8,988,109	10,830,037	11,883,427	3,339,771	35,041,344 (50.0%)
Genes sampled by reads:	10,797	13,384	12,198	13,814	15,701
Poly (A) sites	17,964	27,691	21,431	29,055	48,766
Known poly (A) sites sampled:	12,166	16,958	14,305	17,233	21,406
Putative novel poly (A) sites:	5,798	10,733	7,126	11,822	27,360
Genes sampled by poly (A) sites:	10,397	12,723	11,532	13,041	15,205

* IP: internal priming.

**Figure 2:**
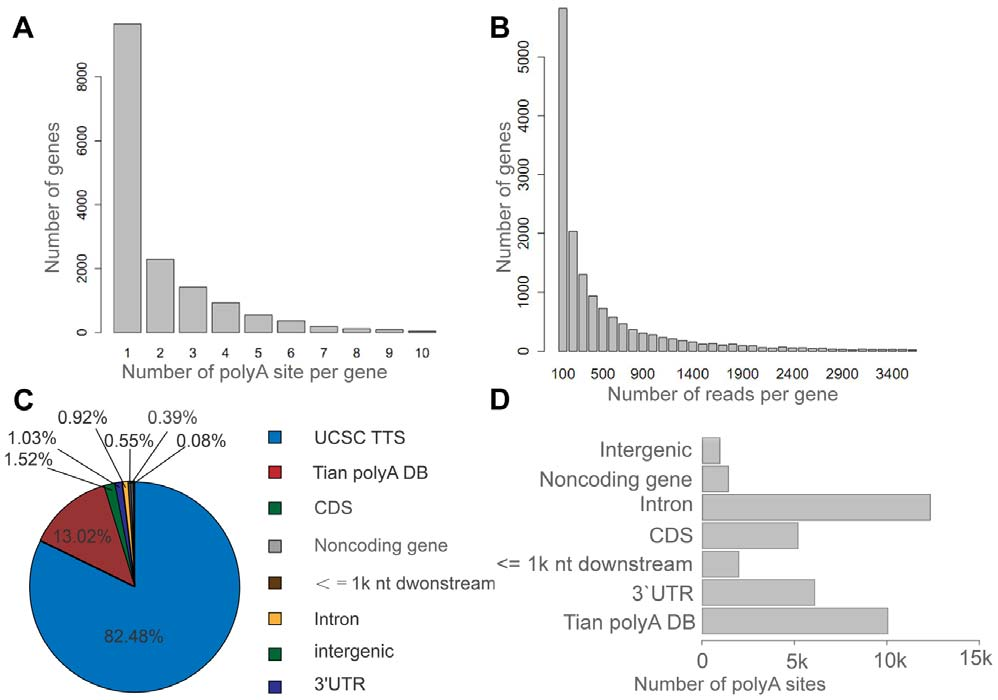
The characteristics of the SAPAS data. A) The distribution of numbers of poly(A) sites per gene. B) The distribution of the number of reads per UCSC canonical gene. C) The genomic locations of the reads that were uniquely mapped to the nuclear genome after internal priming filtering. D) The genomic locations of the poly(A) sites in all genes.

In total, we sequenced 15,205 UCSC canonical genes with at least one read, which accounted for 60% of all canonical genes. Importantly, we also noticed that 5,858 (38.5%) of these genes had more than one tandem APA site, and 3,639 (24%) genes harbored more than two tandem APA sites (Figure 2A). The distribution of the number of all reads is shown in Figure 2B. In addition, our analysis showed that almost all of the filtered reads (95.5%) produced by our research were mapped to known poly(A) sites in the UCSC transcript ends database and Tian's database [7], and an additional 1.03% and 0.6% of the reads were mapped to the 3′UTR and 1 kb downstream from the UCSC canonical genes, respectively (Figure 2C). We identified 48,766 poly(A) sites from our samples. We found approximately 42.9% of these sites in the UCSC and Tian's databases and another 25.3%, 12.4% and 10.9% of the poly(A) sites in the introns, 3′UTRs and CDSs from the UCSC canonical genes, respectively (Figure 2D).

### (3) Differential usage of poly (A) sites between nasal polyp specimens and nasal uncinate process mucosa tissue

**Figure 3:**
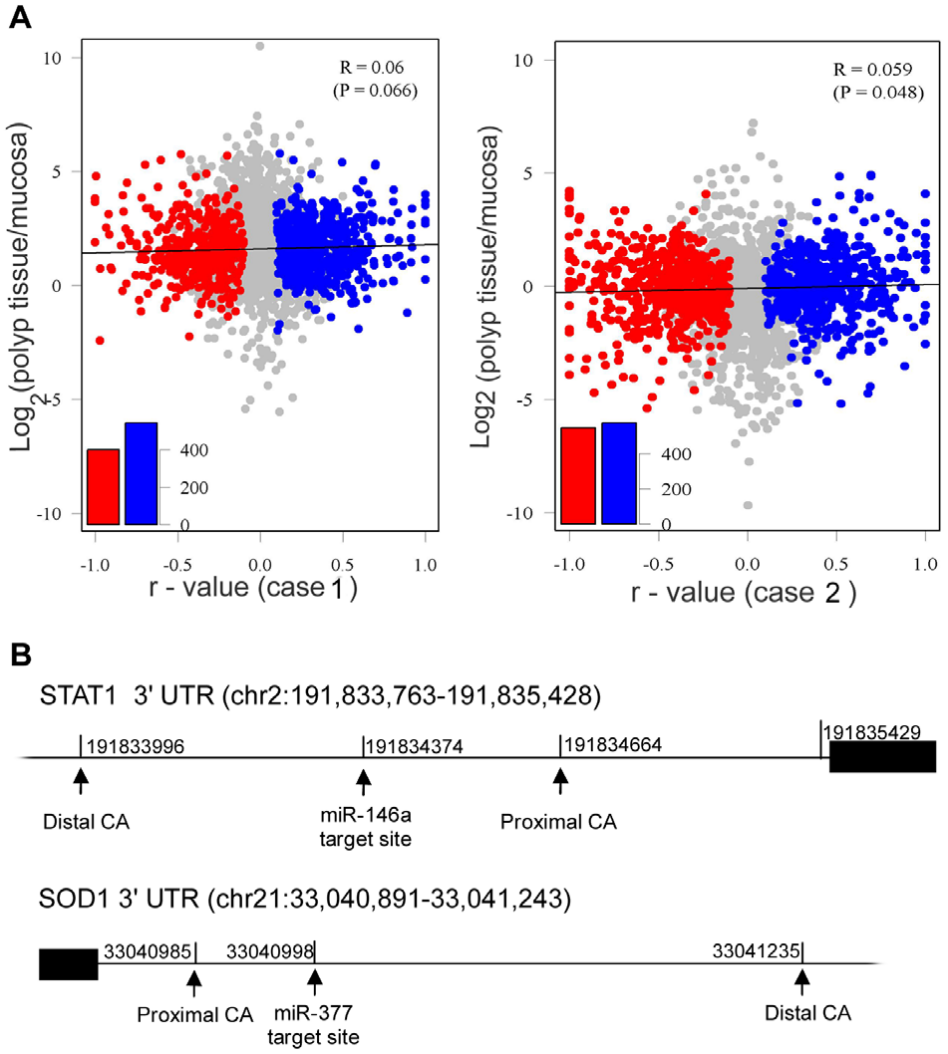
An analysis of APA switching genes. A). APA site switching and gene expression levels of nasal mucosa and nasal polyp. The x-axis denotes the r-value. A larger positive value shows that nasal polyp samples are prone to use longer tandem 3′UTRs. Genes with significant switching to longer (blue) or shorter (red) tandem UTRs in nasal polyp samples (FDR = 0.01; see Methods) are colored. The y-axis denotes the logarithm of the expression levels of genes from the nasal polyp sample corresponding to the nasal mucosa sample. B). Experimentally validated mir-377 and mir-146a target sites in 3′UTR of *SOD1* and *STAT1* mRNA.

Several previous studies discovered that generally highly proliferative cells [9] or cancer cells [10] tend to have shorter 3′UTRs. In this study, we performed a comparison of the tandem 3′UTR lengths of nasal polyp tissue and paired nasal uncinate process mucosa from two patients with CRSwNP using the linear trend alternative to independence test. We denoted the paired nasal uncinate process mucosa as 1 and the nasal polyp tissue as 2 and calculated a Pearson correlation, *r*. A positive *r*-value indicates that the genes in the nasal polyp tissue used longer tandem 3′UTRs than the ones in the paired nasal uncinate process mucosa, and a negative *r*-value indicates that the genes in the nasal polyp tissue used shorter tandem 3′UTRs than the ones in the paired nasal uncinate process mucosa. Based on the *r*-values, we identified 1,033 genes in patient 1 (FDR = 0.01, |*r*|≥0.1) with a significant difference in the tandem 3′UTR length between nasal polyp tissue and the paired nasal uncinate process mucosa and 1,122 genes (FDR = 0.01, |*r*|≥0.1) in patient 2 (Figure 3A). After merging the results of the two cases, we identified 1,948 genes (FDR = 0.01) with a significant difference in the tandem 3′UTR length between nasal polyp tissue and nasal uncinate process mucosa, including 1,016 genes that were switched to mRNA transcripts with longer 3′UTRs in nasal polyp tissue and 932 genes that were switched to mRNA isoforms with shorter 3′UTRs in nasal polyp tissue. Notably, the *r*-values of 48% (932/1,948) of the APA-switching genes were negative. This result indicated that approximately half of all of the identified genes used shorter 3′UTR transcripts. Therefore, there appears to be an equal representation of switching to longer and shorter isoforms. The results implied that the tendency of 3′UTR switching in nasal polyp tissue was different from that of transformed cells or highly proliferative cells, which tend to use shortened 3′UTRs. The inconsistency could be explained by the fact that the formation of nasal polyps in chronic rhinosinusitis appears to be the end result of chronic sinonasal inflammation [13], [14]. In addition, we analyzed a correlation between the 3′UTR length and the gene expression level. For the genes with altered APA sites in our data, we did not observe a positive correlation between the 3′UTR length and the gene expression level (Figure 3A). 3′UTRs are the major target of miRNA-mediated regulation of gene expression. One of the direct effects of 3′UTR length switching may be the gain or loss of miRNA binding sites, which may mediate the stability and translation of mRNA. In this study, we also analyzed two experimentally validated miRNA target sites in two genes that were characterized as APA-switching by our data. As shown in Figure 3B, only the transcripts of the *SOD1* gene with the longer 3′UTR [15], [16] contained the mir-377 target site, and the transcripts of the *STAT1* gene with the shorter 3′UTR lost the mir-146a [17] binding site.

### (4) Functional annotation analysis of the genes with distinct APA site usage

**Figure 4:**
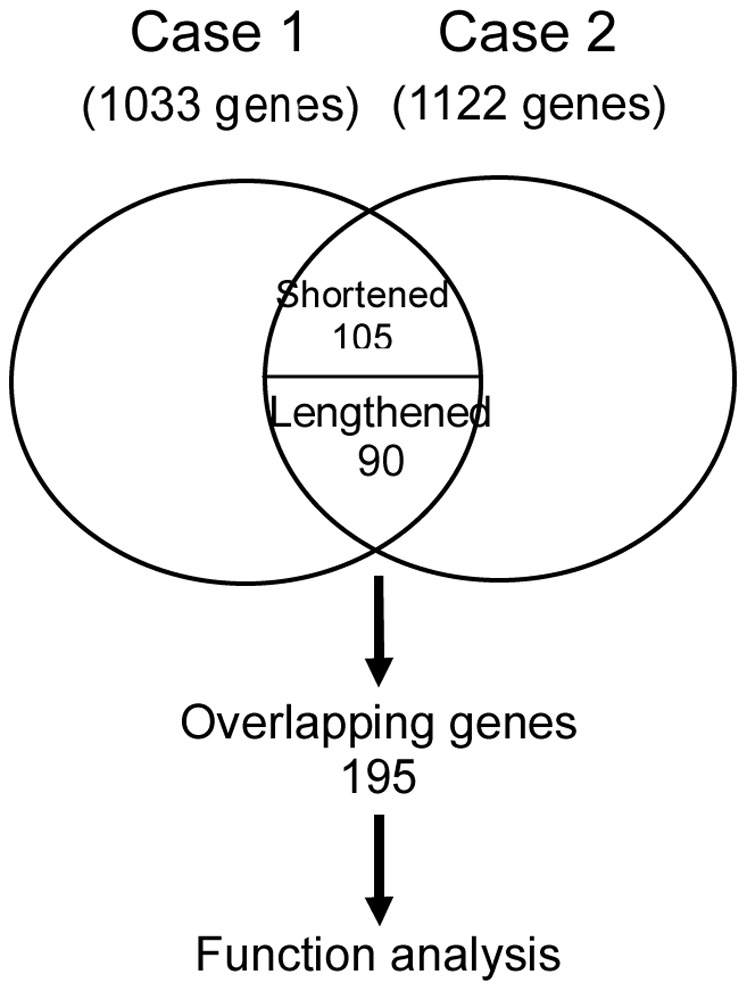
Venn diagram of genes with 3′UTR length changes between the two cases.

To determine the function of these significant APA site-switching events in the formation of nasal polyps, we identified 195 genes that were detected in both patients among the above 1,948 genes, including 105 genes that were likely to use longer 3′UTR isoforms and 90 genes that were likely to use shorter 3′UTR isoforms in nasal polyp tissue (Figure 4). We then performed functional annotation of these genes using the web-accessible DAVID program. The results of Gene Ontology (GO), Pathway, and SP_PIR_Keywords analyses indicated that nine GO terms were significantly enriched in the APA-switching genes (Table 3).

**Table 3:** Enrichment of genes with significantly shorter or longer 3′UTR isoforms involved in various GO functional categories.

GO category		Count	P-value
Shortened genes (90)			
KEGG_PATHWAY	Wnt signaling pathway	4	0.026
GOTERM_BP_FAT	transcription	18	0.037
GOTERM_CC_FAT	nucleolus	12	0.00041
SP_PIR_KEYWORDS	transcription regulation	17	0.023
SP_PIR_KEYWORDS	mRNA splicing	6	0.0029
Lengthened genes (105)			
GOTERM_BP_FAT	apoptosis	12	0.00088
GOTERM_BP_FAT	protein modification by small protein conjugation or removal	6	0.0027
GOTERM_CC_FAT	mitochondrion	13	0.034
SP_PIR_KEYWORDS	mitochondrion	10	0.036

DAVID Bioinformatics Resources 6.7 (http://david.abcc.ncifcrf.gov/) was used to perform a functional annotation.

The reduced apoptosis of inflammatory cells plays a crucial role in the chronic persistence of the inflammatory response associated with the formation of nasal polyps [18]. In the list of genes with longer 3′UTRs, we observed an obvious enrichment of apoptosis-related GO terms (Table 4). Among these genes are *DEDD*, the protein product of which is associated with caspase-8/10, signals cell death, and may be an important mediator of the death receptors [19]; and *p53RFP*, which encodes a p53-inducible E3 ubiquitin ligase that induces p53-dependent but caspase-independent apoptosis [20]. The APA site switching of these genes might contribute to the delay of apoptosis of inflammatory cells, particularly eosinophils, a phenomenon that is of particular interest for further investigations. Also among these genes are *SOD1* and *SOD2*, which are members of the superoxide dismutase gene family that encode antioxidant enzymes responsible for destroying free superoxide radicals in the body; these radicals are normally produced within cells and are toxic to biological systems. Our results indicated that the antioxidant functions of *SOD1* and *SOD2* in nasal polyps are also regulated by the APA site switching of these genes. In the list of genes with shorter 3′UTRs in the nasal polyp tissue, 18 genes are associated with transcription (Table 5), leading to the significant enrichment of transcription–related GO terms. Notably, two genes *(STAT1* and *SAP 30L)* are involved in the IFN-γ and TGF-β signaling pathways, respectively [21], [22]. It is generally accepted that IFN-γ and TGF-β are involved in the pathogenesis of chronic rhinosinusitis with nasal polyps [23], and our results indicated that the transcripts of the two genes with the shorter 3′UTRs might impact the IFN-γ and TGF-β signaling pathways. Additionally, two genes (*ZNF148* and *ZNF384*, also known as *Nuclear matrix transcription factor*) are involved in the transcription of matrix metalloproteinase during extracellular matrix remodeling. Extracellular matrix remodeling is one of the significant characteristics of nasal polyps [24], [25], and our results indicated that the transcripts of the two genes with the shorter 3′UTRs may promote extracellular matrix remodeling of nasal polyp tissue. More interestingly, two genes (*RUNX3* and *ARHGEF17*) can function as tumor suppressor genes [26], [27], and neither of these two genes has been previously investigated in the pathogenesis of nasal polyps. Notably, we found that the genes involved in the Wnt pathway were enriched (P = 0.026; Table 6, Figure S1), and four genes (*FZD5*, *LRP6*, *PPP2R1B*, and *TBL1XR1*) in this pathway switched to proximal APA sites in nasal polyp tissue. This pathway plays an essential role in the transcriptional activation of cell proliferation [28]. Cell proliferation (e.g., of epithelium cells, goblet cells and glandular cells) has been confirmed in nasal polyp tissue [24]. Our results indicated that the APA site switching of these genes might promote the cell proliferation of the nasal polyp tissue.

**Table 4:** Twelve genes enriched in GO terms associated with apoptosis.

ucsc ID	Gene Symbol	Gene Name	r-value
uc003vej.2	BCAP29&	B-cell receptor-associated protein 29	0.95*
uc011jzo.1	TAX1BP1&	Tax1 (human T-cell leukemia virus type I) binding protein 1	0.37*
uc009wty.2	DEDD&	death effector domain containing	0.27*
uc003jqw.3	MAP3K1	mitogen-activated protein kinase kinase kinase 1	0.63*
uc001iia.2	NET1	neuroepithelial cell transforming 1	0.21*
uc003ncs.2	RNF144B	ring finger protein 144B	0.52*
uc001jrm.2	SGPL1	sphingosine-1-phosphate lyase 1	0.52*
uc002ypa.2	SOD1&	superoxide dismutase 1, soluble	0.15*
uc003qsi.1	SOD2	superoxide dismutase 2, mitochondrial	0.53*
uc001bvw.1	YARS	tyrosyl-tRNA synthetase	0.24*
uc002njr.2	UBA52	ubiquitin A-52 residue ribosomal protein fusion product 1	0.19*
uc001aqs.3	UBE4B	ubiquitination factor E4B (UFD2 homolog, yeast)	0.36*

* p<0.001.

& : genes used for q-RT PCR validation.

*r*-value: a positive value of *r* indicates a longer tandem 3′UTR in nasal polypoid tissue, and vice versa.

**Table 5:** Eighteen genes enriched in GO terms associated with transcription.

ucsc ID	Gene Symbol	Name	*r*-value
uc003kyd.2	AFF1	AF4/FMR2 family, member 4	−0.39*
uc003elq.3	CNBP	CCHC-type zinc finger, nucleic acid binding protein	−0.22*
uc010iwg.2	GPBP1	GC-rich promoter binding protein 1	−0.24*
uc010gdb.2	GZF1	GDNF-inducible zinc finger protein 1	−0.97*
uc001lgz.2	IKZF5	IKAROS family zinc finger 5 (Pegasus)	−0.34*
uc003nyk.2	RDBP	RD RNA binding protein	−0.34*
uc003lvk.2	SAP30L	SAP30-like	−0.80*
uc002tpn.2	SAP130	Sin3A-associated protein, 130 kDa	−1*
uc011jxz.1	AHR	aryl hydrocarbon receptor	−0.19*
uc010fyy.2	HDAC4	histone deacetylase 4	−0.58*
uc001bjq.2	RUNX3	runt-related transcription factor 3	−0.89*
uc002usj.2	STAT1	signal transducer and activator of transcription 1, 91 kDa	−0.27*
uc003fiw.3	TBL1XR1	transducin (beta)-like 1 X-linked receptor 1	−0.68*
uc003etw.2	ZBTB38	zinc finger and BTB domain containing 38	−0.87*
uc003ehx.3	ZNF148	zinc finger protein 148	−0.32*
uc001qpz.2	ZNF384	zinc finger protein 384	−0.32*
uc001nmx.3	ZFP91	zinc finger protein 91 homolog (mouse)	−0.30*
uc001otu.2	ARHGEF17	Rho guanine nucleotide exchange factor (GEF) 17	−0.65*

* p<0.001.

*r*-value: a positive value of *r* means longer tandem 3′UTR in nasal polypoid tissue, and vice versa.

**Table 6:** Four genes enriched in the wnt pathway.

ucsc ID	Gene Symbol	Name	r-value
uc002vcj.2	Fzd5	frizzled homolog 5 (Drosophila)	−0.65*
uc001rah.3	LRP1	low density lipoprotein receptor-related protein 6	−0.80*
uc001plx.1	PPP2R2B	protein phosphatase 2 (formerly 2A), regulatory subunit A, beta isoform	−0.98*
uc003fiw.3	TBL1XR1	transducin (beta)-like 1 X-linked receptor 1	−0.70*

* p<0.001.

*r* **-**value: a positive value of *r* indicates a longer tandem 3′UTR in nasal polypoid tissue, and vice versa.

None of these genes has been previously investigated in the pathogenesis of nasal polyps, and the details of the 195 genes (including the GO analysis and gene name) are shown in Table S1and Table S2. In addition to the overlap of these genes between the two samples, the other APA-switching genes in nasal polyp tissue were more prevalent and complex in this GO-term analysis (data not shown).

### (5) Differential gene expression profile analysis between nasal polyps and control tissue

We conducted a gene expression survey of nasal polyp tissue and control nasal mucosa by calculating the number of gene reads produced using Illumina second-generation sequencing. The distribution of the number of reads is shown in Figure 2B. By conducting a pair-wise comparison of the gene expression in the nasal polyp tissue and in the nasal mucosa tissue, we identified 213 genes that were upregulated by at least 3-fold and 414 genes that were downregulated by at least 3-fold in the CRSwNP specimens. We noticed that the GO categories of lymphocyte activation; lymphocyte, leukocyte and mononuclear cell proliferation, defense and inflammation response; activation of innate immune response; and cell cycle phase were enriched in the upregulated genes (P<0.05). In contrast, the GO categories of cell death and apoptosis, negative regulation of protein kinase activity and immune system processes, regulation of microtubule cytoskeleton organization, cell morphogenesis, and skeletal muscle organ development were enriched among the downregulated genes (P<0.05). The details of these genes are shown in Table S3.

### (6) Real-time RT-PCR validation of results

To validate the above two analyses, we performed quantitative real-time RT-PCR. First, we selected 5 genes to use for validation (*c2orf68, Ube2e2, CSK, C8orf84*,and *coq7*) that exhibited extreme 3′UTR length differences between the two samples, and with the exception of *CSK*, the results of all of the genes were confirmed (Table S4). Second, among the 10 differentially expressed genes (*VTCN1, Diablo, srp54, PES1, TACO1, TBRG4, BRPF3, Jhdm1d, skap2, BATF3*) selected at random, the results for 6 genes were consistent with our sequencing data, despite the different rank orders and magnitudes between the two methods (Table S5).

**Figure 5:**
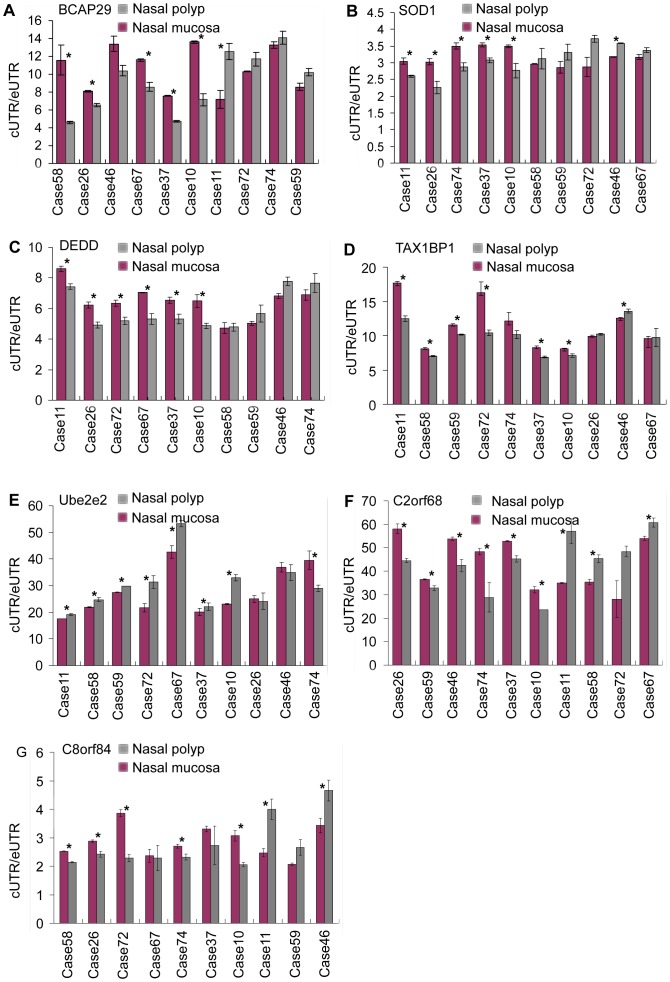
Real-Time PCR analysis in 10 additional patients. A) *BCAP29*. B) *SOD1*. C) *DEDD*. D) *TAX1BP*. E) *c2orf68*. F) *Ube2e2*. G) *C8orf84*. cUTR/eUTR: constitutive UTR/extended UTR, the expression ratios of the shortened region to the lengthened region. *: p<0.05.

Furthermore, to confirm the APA site-switching regulation of the APA-switching genes in nasal polyp, we included an additional 10 patients in the qPCR validation. Moreover, we selected four genes (*BCAP29*, *SOD1*, *DEDD* and *TAX1BP1*) that tended to use longer 3′UTR transcripts and that were enriched in apoptosis-related GO terms, and then we performed quantitative real-time RT-PCR. As Figure 5 shows, in 5–7 patients of the 10 additional cases, *BCAP29*, *SOD1*, *DEDD* and *TAX1BP1* tended to use longer 3′UTR transcripts, similarly to the sequencing data. Additionally, the difference showed a statistically significant p-value (p<0.05). As to the 3 genes (*c2orf68, Ube2e2*, and *C8orf84*), 6–7 patients of the 10 additional cases showed a consistent tendency with the sequencing data. These results further indicated that APA site-switching regulation events were prevalent in nasal polyp tissue.

## Discussion

Recently, many published reports have proposed that changes in 3′UTR length mediated by usage of APA sites are a coordinated mechanism for regulating the expression of genes in various physiological and pathological processes, such as T-cell activation [9], embryonic development [29], cellular transformation [10], tumor cellular proliferation [30], and immune responses [12]. Given that chronic rhinosinusitis with nasal polyps is highly associated with T-cell activation, APA regulation may be associated with this condition. In this study, we compared genome-wide profiling of tandem 3′UTRs in nasal polyp tissue with profiling in the corresponding nasal uncinate process tissues. In these analyses, we identified 1,948 genes (FDR = 0.01) with a significant difference in the tandem 3′UTR length between nasal polyps and nasal uncinate process mucosa, thus linking tandem 3′UTR length switching with the pathologic process of chronic rhinosinusitis with nasal polyps.

3′UTRs are the major target of miRNA-mediated gene expression regulation. Almost all human mRNA transcripts are known to contain more than one miRNA target site, with an average of over 20 miRNA target sites per transcript [31]. An alteration of 3′UTR length must lead to a loss or gain of binding sites [32]. Previous experimental evidence has demonstrated that mir-377 could interact with sequence elements of the 3′UTR of *SOD1* mRNA and influence the level of the protein product of this gene [15], [16]. Our study suggested that the *SOD1* gene typically used distal APA sites and produced mRNAs with longer 3′UTRs in polyp tissue. We noticed that only the transcripts with longer 3′UTRs harbor the binding sites of mir-377. Therefore, only the longer mRNA transcripts can be regulated by mir-377. In contrast, our research also showed that the *STAT1* gene typically used proximal APA sites and produced mRNAs with shorter 3′UTRs in polyp tissue. Experimental evidence has demonstrated that mir-146a can interact with the regulatory elements in transcripts with longer 3′UTRs but not with shorter 3′UTRs [33]; therefore, the shorter 3′UTR transcripts escaped from the control of mir-146a. Although our study has not addressed these questions directly, our study underscores the importance of alternative polyadenylation in the regulation of gene expression in nasal polyps.

In addition to the APA-switching analyses, we also identified 627 genes that were significantly differentially expressed in polyp tissue compared with control tissue. We identified 213 genes that were up-regulated by at least 3-fold in CRS polyps. These genes were involved in various cellular biological functions, including lymphocyte activation; lymphocyte, leukocyte and mononuclear cell proliferation; defense and inflammatory responses; activation of the innate immune response; and cell cycle phase regulation. This result was not surprising because it has been confirmed that immune and inflammatory reactions play a pivotal role in the development and maintenance of nasal polyps [14]. Additionally, our observations also indicated that the down-regulated genes in nasal polyps were mainly associated with apoptosis and cell death as well as the negative regulation of protein kinase activity. This observation was confirmed in another study by Qiu [34] et al., who reported the overexpression of the *BIRC5* gene, a novel member of the group of inhibitors of apoptosis proteins, in nasal polyps from patients. Most importantly, these results suggested that our experiments effectively detected the transcripts of nasal polyps and control tissues that were involved in the pathogenesis of nasal polyps.

In conclusion, APA site-switching events in 3′UTRs are prevalent in nasal polyp tissue, and the regulation of gene expression by APA may play an important role in the formation and maintenance of nasal polyps. The genes that were identified to undergo APA site-switching events included transcription factors, regulators of cell proliferation and apoptosis, members of cytokine signaling pathways, and growth factors/receptors. Novel therapeutic interventions targeting the APA site-switching events of these genes might produce tangible clinical effects.

## Materials and Methods

### Ethics statement

This study was conducted under institutional approval from the local research ethical committee (the Internal Review and the Ethics Boards of the Sun Yat-sen Memorial Hospital, Sun Yat-sen University). Informed written consent was provided by all participants.

### Evaluation of patients

Twelve patients suffering from CRS with eosinophil-rich nasal polyps who were treated surgically at the Department of Otorhinolaryngology of Sun Yat-sen Memorial Hospital were included in this study group. Nasal polyp tissues and the corresponding mucosa of the uncinate process were sampled for this research. Samples from 2 patients were used for SAPAS sequencing, and the other samples from the remaining 10 patients were used for real-time PCR. Patients with an established immunodeficiency, allergic fungal sinusitis, ciliary dyskinesias, sinonasal tumor, atopy or cystic fibrosis were excluded from the study. The clinical data of every patient are shown in Table 1.

The degree or strength of individual rhinosinusitis symptoms (nasal obstruction, anterior and posterior nasal discharge, smell abnormalities, and facial pain and pressure) was recorded as severe, moderate, slight or no symptom [35]. The extent of disease was assessed by computed tomography (CT) scanning and nasal endoscopic testing. The polyps were graded by size and extent in both the left and right nasal fossa on a scale of 0–3, according to the Davos classification [36]. The findings on the CT scans were graded according to the Lund–Mackay score [36]. The diagnosis of chronic rhinosinusitis with nasal polyps was based on history, clinical manifestations, nasal endoscopy, and computed tomography (CT) scan of the sinuses according to the EP^3^OS guidelines [35]. Eosinophilic polyp patients were identified histologically by counting the number of eosinophils at 200× magnification under light microscopy through histological examination after the operation [11]. Five fields were examined for each section and the average was considered to be the number of eosinophils infiltrating the sample [11]. Atopy was defined by a positive personal history of allergic respiratory symptoms and positive skin prick tests (SPT; wheal>3 mm) to the standard panel of inhalant allergens. Evaluation of asthma was performed according to the Global Initiative on Asthma (GINA) [37].

All of the patients with nasal polyps (NPs) received intranasal glucocorticoid therapy for more than one year but failed to respond to medical treatment and accordingly underwent endoscopic sinus surgery. Importantly, the patients had normal-appearing mucosal tissue of the uncinate process that was to be excised during the surgery.

### RNA extraction

The nasal polyp tissue and paired nasal uncinate process tissue removed during endoscopic sinus surgery were submerged in the RNAlater® reagent (Qiagen, Valentia, CA) to avoid RNA degradation, and the samples were preserved in a −80°C refrigerator for subsequent RNA extraction. Total RNA was extracted using the TRIzol reagent (Invitrogen, Carlsbad, CA) according to the manufacturer's instructions. The quality of the extracted RNA was analyzed using electrophoresis in a 1.5% agarose gel stained with ethidium bromide. The quantity of the extracted RNA was determined spectrophotometrically using a NanoDrop 1000 spectrophotometer (Nano-Drop Technologies, Wilmington, DE, USA). The RNA purity was assessed by the ratio of absorbance at 260 and 280 nm (A260/A280) (ratios between 1.9 and 2.1 were acceptable). The extracted RNA was digested with RNase-free DNase (Toyobo, Osaka, Japan) and purified with a mini-spin column using an RNeasy Mini Total RNA Purification Kit (Qiagen, Valencia, CA).

### Preparation of the 3′UTR library and Illumina sequencing

The SAPAS sequencing libraries were constructed as previously described [12]. Briefly, total RNA was randomly fragmented by heating. Using template-switching technology and an improved reverse transcription (RT) reaction mixture, high-quality 3′-anchored first-strand cDNA was generated with Super Script II reverse transcriptase (Invitrogen Life Technologies, Karlsruhe, Germany). Concurrently, a 5′template-switching adaptor tagged with Illumina adaptors was added (Table S6). Next, ds-cDNA was synthesized by PCR amplification with known sequencing primers and Platinum® Taq DNA Polymerase High Fidelity (Invitrogen, Carlsbad, CA, USA). Fragments of 300–500 bp were selected from the PCR products by performing PAGE separation, excision, and gel extraction with a QIAquick Gel Extraction Kit (Qiagen, Valencia, CA). The final pooled fragments were sequenced from the 3′end with an Illumina Solexa GA IIx (Illumina, San Diego, CA). These sequencing data were uploaded to the MIAME compliant Gene Expression Omnibus (GEO) database at National Center for Biotechnology Information (http://www.ncbi.nlm.nih.gov/geo) and is accessible through accession number GSE39957).

### Bioinformatic methods

1. **Filtering and mapping of Illumina reads**: The filtering and trimming of all of the reads were performed with Perl scripts. Those reads were discarded if they did not begin with the linker 5′-TTTTCTTTTTTCTTTTTT-3′ or if their length was less than 25 nt. Next, the linker was trimmed, as were the “T”s that followed the linker, until a not-“T” (i.e., an A, C or G, but not an N) was encountered. All of the remaining reads were aligned to the human genome (hg19; downloaded at UCSC genome bioinformatics [39]) with Bowtie [38] (version 0.12.5; parameters: -q -p 5 -k 2 –best -v 2), allowing for 2 mismatches. The uniquely mapped reads were used for internal priming filtering by examining the genomic sequence 1 to 20 bases downstream of poly(A) cleavage sites. The uniquely mapped reads were considered to be internal priming candidates and then removed if they contained more than 12 “A”s or one of the following patterns: 5′-AAAAAAAA-3′ and 5′-GAAAA+GAAA+G-3′ (in which “+” means “or more”) in the 20 nt region immediately downstream from poly(A) cleavage sites.
2. **Clustering of reads and identification of poly(A) sites:** All of the reads of the samples were iteratively clustered as described previously, and then the poly(A) cleavage sites that are located next to each other within 24 nt were clustered. Next, cleavage clusters with two or more reads were assigned as poly(A) sites.
3. **Tandem 3′UTR annotation:** For annotation of 3′UTRs, first a dataset of all known 3′UTR regions was extracted from the Known Genes database of the UCSC table browser [39], as follows: 1) neglect all noncoding gene items; 2) consider only the last exon for each item in knownGenes; and 3) take the stop codon (if one was present in the last exon) or the 5′end of the last exon (if no stop codon existed in the last exon) as the beginning of the 3′UTR and take the 3′end of the last exon as the end of the 3′UTR for each knownGenes item. A poly(A) site was defined as a tandem poly(A) site if 1) the 5′end of the corresponding cleavage cluster was located within one known 3′UTR region and only one item of known 3′UTR regions was defined above; 2) the corresponding cleavage cluster was located within 1 kb downstream of one and only one item of known 3′UTR regions and contained a read that overlapped a particular item of known 3′UTR regions; 3) the cleavage cluster was located within 1 kb downstream of one and only one item of known 3′UTR regions and contained a read that overlapped one of the tandem poly(A) sites that belonged to the corresponding item of known 3′UTR regions. If two or multiple poly(A) sites are found in a gene, then the gene has a tandem 3′UTR.
4. **Comparison of tandem 3′UTR switching between samples:** We tested tandem 3′UTR switching events among different samples through adopting a method to test linear trend alternative to independence for two-way tables with ordered classifications [40]. For a co-expressed (in both polyp tissue and mucosa tissue) UCSC gene with two or more tandem poly(A) sites, we performed the test using the following steps: 1) calculate the tandem UTR length for each tandem poly(A) site; 2) list the number of reads for each tandem poly(A) site for each sample in a table: take the tandem poly(A) sites as columns (from the site with the shortest UTR to that with the longest) and take the two samples as rows (mucosa tissue sample and polyp tissue); 3) if the total number of reads in the table is less than 30, neglect this gene for the test; 4) let the lengths of the tandem UTRs denote the scores for the columns; let 1 denote the row score for the mucosa tissue sample and let 2 denote the row score for the nasal polyp tissue; 5) calculate the Pearson correlation 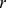 using the number of reads in the table as the values and using the scores for the rows and columns as coordinates; 6) calculate a statistic: *M^2^ = (n−1)r^2^*; for large samples, this statistic is approximately chi-squared with df = 1, and a P-value can be obtained. The Benjamini-Hochberg FDR was estimated using the R software. Moreover, tandem 3′UTR length switching with significant P-values paired to a false discovery rate cutoff of 1% were considered to be significantly different between the two samples. A positive value of *r* indicates a longer tandem 3′UTR in nasal polyp tissue and vice versa.
5. **Functional annotation analysis of the genes with switched APA sites**: Functional annotation of the detected overlapped genes between the two patients was performed using the DAVID Bioinformatics Resources (http://david.abcc.ncifcrf.gov/) [41]. We searched for significantly enriched Biological Process GO terms, pathways, and SP_PIR_Keywords against a background model of all transcripts found in both polyp tissue and control mucosa.

### Validation of qRT-PCR analysis

Five genes (*c2orf68, Ube2e2, CSK, C8orf84*, and *coq7*) with extreme 3′UTR length differences between nasal mucosa and nasal polyp tissue, 4 genes (*BCAP29*, *SOD1*, *DEDD* and *TAX1BP1*) enriched in GO terms associated with apoptosis and 10 differentially expressed genes (*VTCN1, Diablo, srp54, PES1, TACO1, TBRG4, BRPF3, Jhdm1d, skap2, BATF3*) were subjected to qRT-PCR to validate the sequencing data. Total RNA was isolated using the TRIzol reagent (Invitrogen, Carlsbad, CA) according to the manufacturer's instructions. For each sample, 100 ng of total RNA was used in reverse transcription reactions using oligo-dT primers and SuperScript III Reverse Transcriptase (Invitrogen, Carlsbad, CA). For each gene, two gene-specific primer sets were designed based on the SAPAS data, one “constitutive” set targeting the regions upstream of the proximal sites, which were shared by the long and the short isoforms, and the other “extended” set targeting the fragments upstream of the distal sites, which were only used by the alternative isoforms (Table S7, Figure S2). The qRT-PCR was performed using the Light Cycler 480 instrument (Roche Biochemicals, Indianapolis, IN, USA) with THUNDER BIRD™ SYBR qPCR Mix (TOYOBO, Kita, Osaka, Japan) according to the manufacturer's instructions. The expression ratios of the shortened region to the lengthened region (cUTR/eUTR) were maintained through calculating ΔΔCt values for each gene by normalizing the extended set against the constitutive one. Significantly differential usage of poly(A) sites of genes between samples was detected by Student's t-test at a significant level of 0.05. For differentially expressed genes, the relative quantification method was used to measure the levels of the genes in nasal polyps, which were normalized to β-actin as an endogenous control.

## Supporting Information

Figure S1 **Enriched Wnt pathway genes that switched to shorter 3′UTRs in nasal polyp tissue.** The genes that switched to longer 3′UTRs are indicated with a star. The figure was modified from the KEGG database.
(DOCX)

Figure S2 **The visual representation of the location of PCR primers of the two genes (CSK and c2orf68).**
(DOCX)

Table S1 **The enrichment of Gene Ontology terms among genes with switched APA sites switched (FDR = 0.01) between nasal polyp and control tissue.** The numbers in parentheses indicate the number of genes, and the numbers after the parentheses indicate the percentage of genes for a particular category.
(DOCX)

Table S2 **APA-switching gene names, corresponding** ***r*** **-value and APA site-switching information.**
(XLS)

Table S3 **The enrichment of Gene Ontology Biological Process terms among differentially expressed genes (greater than 3-fold difference) between nasal polyp tissue and control mucosa tissue.** The numbers in and after the parentheses indicate the number of genes and the percentage for a particular category, respectively.
(DOCX)

Table S4 **Validation of 3′UTR switching in nasal polyp tissue compared with control tissue using RT-PCR.** ER: Expression ratios of the shortened region to the lengthened region. P: polyp tissue; C: control tissue. Pearson *r*: A larger positive/negative value indicates that longer/shorter tandem UTRs are prone to be used in the nasal polyp.
(DOCX)

Table S5 **Validation of differentially expressed genes in nasal polyp tissue and control tissue using qRT-PCR.**
(DOCX)

Table S6 **Primers used for the SAPAS library construction.**
(DOCX)

Table S7 **PCR primers used in qRT-PCR.**
(DOCX)

## References

[1] Hamilos DL (2011) Chronic rhinosinusitis: Epidemiology and medical management. J Allergy Clin Immunol 128: 693–707. 21890184 10.1016/j.jaci.2011.08.004

[2] Rostkowska-Nadolska B, Kapral M, Fraczek M, Kowalczyk M, Gawron W, et al (2011) A microarray study of gene expression profiles in nasal polyps. Auris Nasus Larynx 38: 58–64. 20554417 10.1016/j.anl.2010.05.002

[3] Zander KA, Saavedra MT, West J, Scapa V, Sanders L, et al (2009) Protein microarray analysis of nasal polyps from aspirin-sensitive and aspirin-tolerant patients with chronic rhinosinusitis E-2960-2010. Am J Rhinol Allergy 23: 268–272. 19490800 10.2500/ajra.2009.23.3314

[4] Sekigawa T, Tajima A, Hasegawa T, Hasegawa Y, Inoue H, et al (2009) Gene-expression profiles in human nasal polyp tissues and identification of genetic susceptibility in aspirin-intolerant asthma C-1053-2008. Clin Exp Allergy 39: 972–981. 19489917 10.1111/j.1365-2222.2009.03229.x

[5] Jian W, Liu B, He J, Fan JP (2009) Gene expression profiles of nasal polyps associated with allergic rhinitis. Am J Otolaryngol 30: 24–32. 19027509 10.1016/j.amjoto.2008.01.003

[6] Proudfoot NJ (2011) Ending the message: poly(A) signals then and now. Genes Dev 25: 1770–1782. 21896654 10.1101/gad.17268411 PMC3175714

[7] Tian B, Hu J, Zhang H, Lutz CS (2005) A large-scale analysis of mRNA poly(A)denylation of human and mouse genes. Nucleic Acids Res 33: 201–212. 15647503 10.1093/nar/gki158 PMC546146

[8] Di Giammartino DC, Nishida K, Manley JL (2011) Mechanisms and consequences of alternative poly(A)denylation. Mol Cell 43: 853–866. 21925375 10.1016/j.molcel.2011.08.017 PMC3194005

[9] Sandberg R, Neilson JR, Sarma A, Sharp PA, Burge CB (2008) Proliferating cells express mRNAs with shortened 3′ untranslated regions and fewer microRNA target sites. Science 320: 1643–1647. 18566288 10.1126/science.1155390 PMC2587246

[10] Mayr C, Bartel DP (2009) Widespread Shortening of 3′ UTRs by Alternative Cleavage and Poly(A)denylation Activates Oncogenes in Cancer Cells. CELL 138: 673–684. 19703394 10.1016/j.cell.2009.06.016 PMC2819821

[11] Yoshifuku K, Matsune S, Ohori J, Sagara Y, Fukuiwa T, et al (2007) IL-4 and TNF-alpha increased the secretion of eotaxin from cultured fibroblasts of nasal polyps with eosinophil infiltration. Rhinology 45: 235–241. 17956026

[12] Fu Y, Sun Y, Li Y, Li J, Rao X, et al (2011) Differential genome-wide profiling of tandem 3′ UTRs among human breast cancer and normal cells by high-throughput sequencing. Genome Res 21: 741–747. 21474764 10.1101/gr.115295.110 PMC3083091

[13] Van Drunen CM, Mjosberg JM, Segboer CL, Cornet ME, Fokkens WJ (2012) Role of innate immunity in the pathogenesis of chronic rhinosinusitis: progress and new avenues. Curr Allergy Asthma Rep 12: 120–126. 22311575 10.1007/s11882-012-0249-4 PMC3296037

[14] Van Crombruggen K, Zhang N, Gevaert P, Tomassen P, Bachert C (2011) Pathogenesis of chronic rhinosinusitis: Inflammation. J Allergy Clin Immunol 128: 728–732. 21868076 10.1016/j.jaci.2011.07.049

[15] Milani P, Gagliardi S, Cova E, Cereda C (2011) SOD1 Transcriptional and Posttranscriptional Regulation and Its Potential Implications in ALS. Neurol Res Int 2011: 458427. 21603028 10.1155/2011/458427 PMC3096450

[16] Wang Q, Wang Y, Minto AW, Wang J, Shi Q, et al (2008) MicroRNA-377 is up-regulated and can lead to increased fibronectin production in diabetic nephropathy. FASEB J 22: 4126–4135. 18716028 10.1096/fj.08-112326 PMC2614610

[17] Lu LF, Boldin MP, Chaudhry A, Lin LL, Taganov KD, et al (2010) Function of miR-146a in controlling Treg cell-mediated regulation of Th1 responses. Cell 142: 914–929. 20850013 10.1016/j.cell.2010.08.012 PMC3049116

[18] Simon HU, Yousefi S, Schranz C, Schapowal A, Bachert C, et al (1997) Direct demonstration of delayed eosinophil apoptosis as a mechanism causing tissue eosinophilia. J Immunol 158: 3902–3908. 9103460

[19] Alcivar A, Hu S, Tang J, Yang X (2003) DEDD and DEDD2 associate with caspase-8/10 and signal cell death. Oncogene 22: 291–297. 12527898 10.1038/sj.onc.1206099

[20] Huang J, Xu LG, Liu T, Zhai Z, Shu HB (2006) The p53-inducible E3 ubiquitin ligase p53RFP induces p53-dependent apoptosis. FEBS Lett 580: 940–947. 16427630 10.1016/j.febslet.2005.09.105

[21] Lindfors K, Viiri KM, Niittynen M, Heinonen TY, Maki M, et al (2003) TGF-beta induces the expression of SAP30L, a novel nuclear protein. BMC Genomics 4: 53. 14680513 10.1186/1471-2164-4-53 PMC319701

[22] Schindler C, Fu XY, Improta T, Aebersold R, Darnell JJ (1992) Proteins of transcription factor ISGF-3: one gene encodes the 91-and 84-kDa ISGF-3 proteins that are activated by interferon alpha. Proc Natl Acad Sci U S A 89: 7836–7839. 1502203 10.1073/pnas.89.16.7836 PMC49806

[23] Otto BA, Wenzel SE (2008) The role of cytokines in chronic rhinosinusitis with nasal polyps. Curr Opin Otolaryngol Head Neck Surg 16: 270–274. 18475084 10.1097/MOO.0b013e3282fb2885

[24] Ardehali MM (2011) Histopathologic characteristics of inferior turbinate vs ethmoidal polyp in chronic rhinosinusitis. pp. 233–236. 10.1016/j.anndiagpath.2010.10.009 21396869

[25] Watelet JB, Bachert C, Claeys C, Van Cauwenberge P (2004) Matrix metalloproteinases MMP-7, MMP-9 and their tissue inhibitor TIMP-1: expression in chronic sinusitis vs nasal polyposis. Allergy 59: 54–60. 14674934 10.1046/j.1398-9995.2003.00364.x

[26] Bloethner S, Mould A, Stark M, Hayward NK (2008) Identification of ARHGEF17, DENND2D, FGFR3, and RBI Mutations in Melanoma by Inhibition of Nonsense-Mediated mRNA Decay E-3542-2010. Genes Chromosomes Cancer 47: 1076–1085. 18677770 10.1002/gcc.20598

[27] Soong R, Shah N, Peh BK, Chong PY, Ng SS, et al (2009) The expression of RUNX3 in colorectal cancer is associated with disease stage and patient outcome. Br J Cancer 100: 676–679. 19223906 10.1038/sj.bjc.6604899 PMC2653772

[28] Katanaev VL (2010) The Wnt/Frizzled GPCR signaling pathway. Biochemistry (Mosc) 75: 1428–1434. 21314612 10.1134/s0006297910120023

[29] Ji Z, Lee JY, Pan ZH, Jiang BJ, Tian B (2009) Progressive lengthening of 3′ untranslated regions of mRNAs by alternative poly(A)denylation during mouse embryonic development. Proc Natl Acad Sci U S A 106: 7028–7033. 19372383 10.1073/pnas.0900028106 PMC2669788

[30] Bhaskaran N, Lin KW, Gautier A, Woksepp H, Hellman U, et al (2009) Comparative proteome profiling of MCF10A and 184A1 human breast epithelial cells emphasized involvement of CDK4 and cyclin D3 in cell proliferation. Proteomics Clin Appl 3: 68–77. 21136936 10.1002/prca.200800045

[31] Arnold M, Ellwanger DC, Hartsperger ML, Pfeufer A, Stumpflen V (2012) Cis-acting polymorphisms affect complex traits through modifications of microRNA regulation pathways. PLoS One 7: e36694. 22606281 10.1371/journal.pone.0036694 PMC3350471

[32] Lutz CS, Moreira A (2011) Alternative mRNA poly(A)denylation in eukaryotes: an effective regulator of gene expression. Wiley Interdiscip Rev RNA 2: 22–31. 21956967 10.1002/wrna.47

[33] Lu LF, Boldin MP, Chaudhry A, Lin LL, Taganov KD, et al (2010) Function of miR-146a in controlling Treg cell-mediated regulation of Th1 responses. Cell 142: 914–929. 20850013 10.1016/j.cell.2010.08.012 PMC3049116

[34] Qiu ZF, Han DM, Zhang L, Zhang W, Fan EZ, et al (2008) Expression of survivin and enhanced polypogenesis in nasal polyps. Am J Rhinol 22: 106–110. 18336724 10.2500/ajr.2008.22.3139

[35] Fokkens W, Lund V, Mullol J (2007) EP3OS 2007: European position paper on rhinosinusitis and nasal polyps 2007. A summary for otorhinolaryngologists. Rhinology 45: 97–101. 17708455

[36] Lund VJ, Kennedy DW (1997) Staging for rhinosinusitis. Otolaryngol Head Neck Surg 117: S35–S40. 9334786 10.1016/S0194-59989770005-6

[37] Olaguibel JM, Quirce S, Julia B, Fortuna AM, Molina J, et al (2012) Measurement of asthma control according to 2006 Global Initiative on Asthma Guidelines: MAGIC Study. Respir Res 13: 50. 22726416 10.1186/1465-9921-13-50 PMC3462124

[38] Langmead B, Trapnell C, Pop M, Salzberg SL (2009) Ultrafast and memory-efficient alignment of short DNA sequences to the human genome. Genome Biol 10: R25. 19261174 10.1186/gb-2009-10-3-r25 PMC2690996

[39] Rhead B, Karolchik D, Kuhn RM, Hinrichs AS, Zweig AS, et al (2010) The UCSC Genome Browser database: update 2010. Nucleic Acids Res 38: D613–D619. 19906737 10.1093/nar/gkp939 PMC2808870

[40] Agresti A (2002) Categorical data analysis. New York: Wiley-Interscience.

[41] Huang DW, Sherman BT, Lempicki RA (2009) Systematic and integrative analysis of large gene lists using DAVID bioinformatics resources. Nat Protoc 4: 44–57. 19131956 10.1038/nprot.2008.211

