# Supplementary material for: A Global Analysis of Tandem 3′UTRs in Eosinophilic Chronic Rhinosinusitis with Nasal Polyps
Source: PLoS One. 2012 Nov 19;7(11):e48997. doi: 10.1371/journal.pone.0048997 (PMC3501494; doi:10.1371/journal.pone.0048997)

**Figure S1 Enriched Wnt pathway in genes switched to shorter 3′UTRs in nasal polyp tissue.** The genes switched to longer 3’UTR were stared. The figure was modified from KEGG.


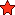

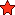

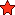

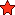

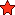

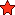

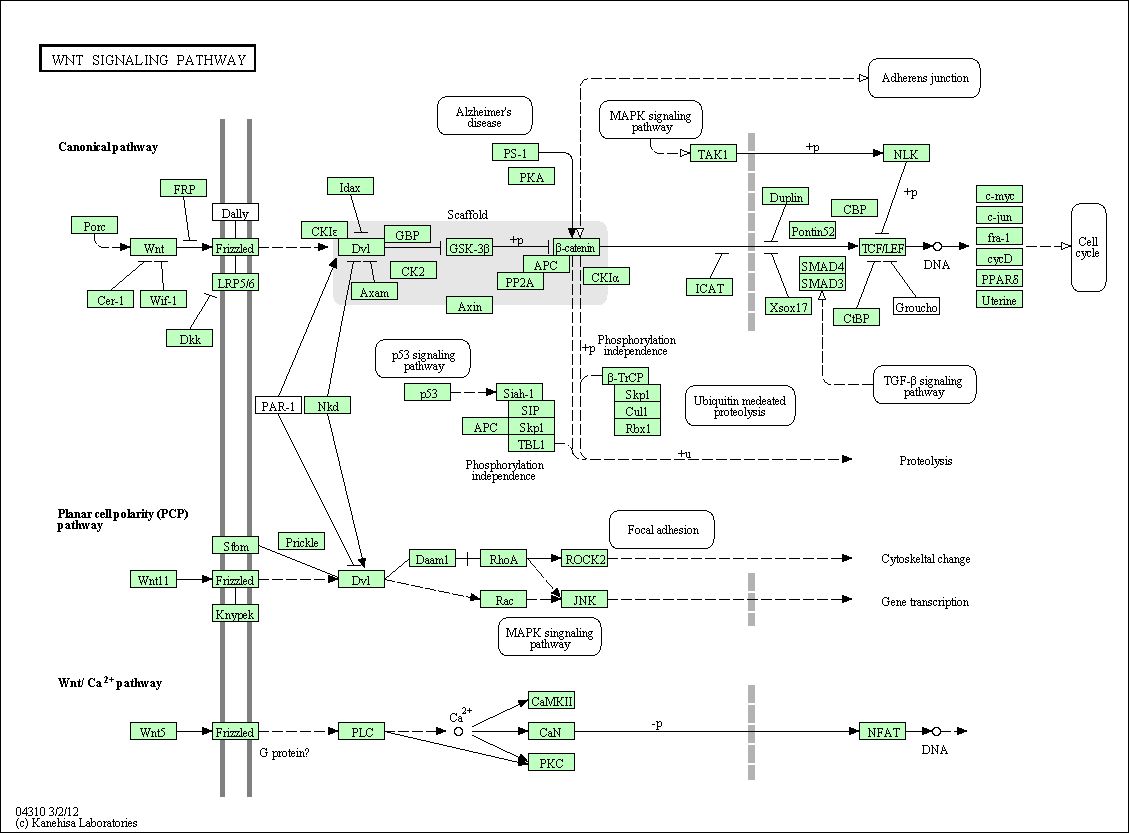

Supplement: Figure S1 — Enriched Wnt pathway genes that switched to shorter 3′UTRs in nasal polyp tissue. The genes that switched to longer 3′UTRs are indicated with a star. The figure was modified from the KEGG database. (DOCX) [file pone.0048997.s001.docx]
