# Supplementary figures and images for: A Global Analysis of Tandem 3′UTRs in Eosinophilic Chronic Rhinosinusitis with Nasal Polyps
Source: PLoS One. 2012 Nov 19;7(11):e48997. doi: 10.1371/journal.pone.0048997 (PMC3501494; doi:10.1371/journal.pone.0048997)

**Figure S2: Visual representations of the location of primers of the two genes (CSK, C2orf68 ).**

**CSK:**


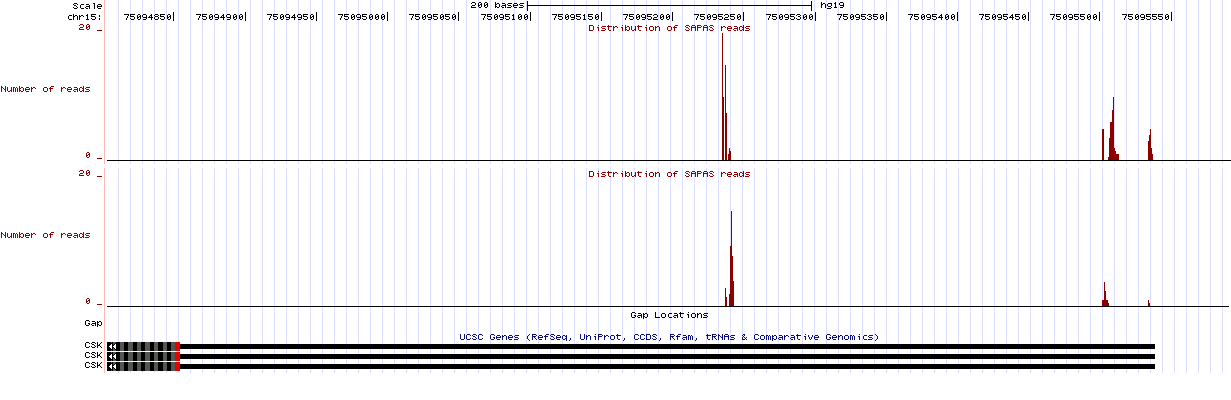


**C2orf68**


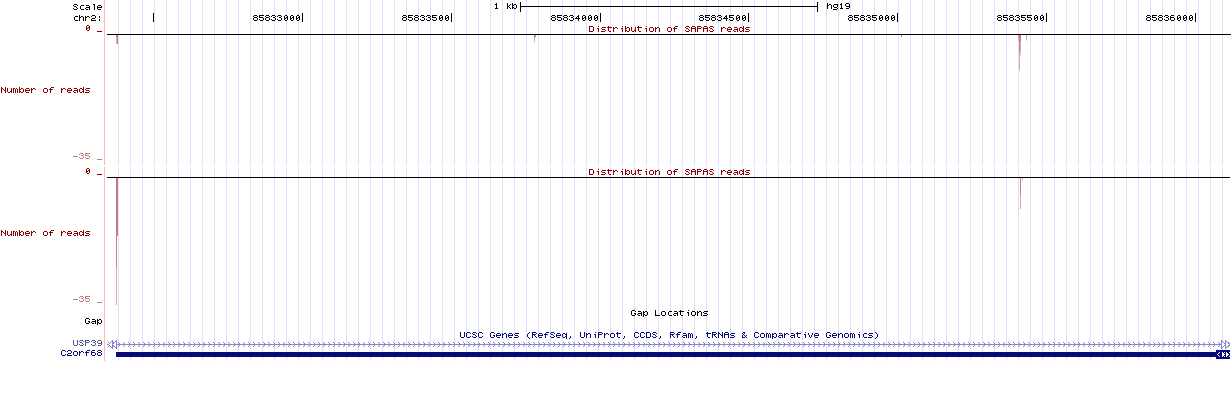

Supplement: Figure S2 — The visual representation of the location of PCR primers of the two genes (CSK and c2orf68). (DOCX) [file pone.0048997.s002.docx]
