# Supplementary material for: A Global Analysis of Tandem 3′UTRs in Eosinophilic Chronic Rhinosinusitis with Nasal Polyps
Source: PLoS One. 2012 Nov 19;7(11):e48997. doi: 10.1371/journal.pone.0048997 (PMC3501494; doi:10.1371/journal.pone.0048997)
Supplement: Table S1 — The enrichment of Gene Ontology terms among genes with switched APA sites switched (FDR = 0.01) between nasal polyp and control tissue. The numbers in parentheses indicate the number of genes, and the numbers after the parentheses indicate the percentage of genes for a particular category. (DOCX) [file pone.0048997.s003.docx]

**Table S1. Enrichment of Gene Ontology in APA sites switched genes (FDR=0.01) between nasal polyp and control tissue.** Numbers in parentheses and ones after it indicate number and percentage of genes for a particular category.

**(1) Genes switched to shorter 3’UTR**

| GO category | Genes switched to shorter 3’UTR (90) |
| --- | --- |
| P<0.05  KEGG_PATHWAY | Sublist Category Term (Count) % P-Value  KEGG_PATHWAY Wnt signaling pathway(4) 4.4% 2.6E-2 |
| P<0.05  GOTERM_BP_FAT | Sublist Category Term (Count) % P-Value  GOTERM_BP_FAT ribonucleoprotein complex assembly(4) 4.4% 5.4E-3  GOTERM_BP_FAT RNA splicing, via transesterification reactions with bulged adenosine as nucleophile(5) 5.6% 8.1E-3  GOTERM_BP_FAT nuclear mRNA splicing, via spliceosome(5) 5.6% 8.1E-3  GOTERM_BP_FAT RNA splicing, via transesterification reactions(5) 5.6% 8.1E-3  GOTERM_BP_FAT ribonucleoprotein complex biogenesis(5) 5.6% 1.4E-2  GOTERM_BP_FAT RNA splicing(6) 6.7% 1.6E-2  GOTERM_BP_FAT mRNA processing(6) 6.7% 2.5E-2  GOTERM_BP_FAT Golgi vesicle transport(4) 4.4% 3.0E-2  GOTERM_BP_FAT transcription from RNA polymerase II promoter(5) 5.6% 3.3E-2  GOTERM_BP_FAT regulation of translation(4) 4.4% 3.4E-2  GOTERM_BP_FAT transcription(18) 20.0% 3.7E-2  GOTERM_BP_FAT positive regulation of RNA metabolic process(7) 7.8% 3.8E-2  GOTERM_BP_FAT mRNA metabolic process(6) 6.7% 4.2E-2  GOTERM_BP_FAT positive regulation of transcription from RNA polymerase II promoter(6) 6.7% 4.3E-2 |
| P<0.1  GOTERM_BP_FAT | Sublist Category Term (Count) % P-Value  GOTERM_BP_FAT intracellular transport(8) 8.9% 5.3E-2  GOTERM_BP_FAT myoblast fusion(2) 2.2% 5.5E-2  GOTERM_BP_FAT syncytium formation by plasma membrane fusion(2) 2.2% 6.0E-2  GOTERM_BP_FAT transcription, DNA-dependent(5) 5.6% 6.4E-2  GOTERM_BP_FAT RNA biosynthetic process(5) 5.6% 6.7E-2  GOTERM_BP_FAT syncytium formation(2) 2.2% 7.0E-2  GOTERM_BP_FAT myotube differentiation(2) 2.2% 8.0E-2  GOTERM_BP_FAT Wnt receptor signaling pathway through beta-catenin(2) 2.2% 8.4E-2  GOTERM_BP_FAT establishment of RNA localization(3) 3.3% 9.0E-2  GOTERM_BP_FAT RNA transport(3) 3.3% 9.0E-2  GOTERM_BP_FAT nucleic acid transport(3) 3.3% 9.0E-2  GOTERM_BP_FAT RNA localization(3) 3.3% 9.5E-2  GOTERM_BP_FAT posttranscriptional regulation of gene expression (6 ) 6.7% 9.8E-2 |
| GOTERM_CC_FAT P<0.05 | Sublist Category Term (Count) % P-Value  GOTERM_CC_FAT nucleolus(12) 13.3% 4.1E-4  GOTERM_CC_FAT nuclear lumen(16) 17.8% 2.8E-3  GOTERM_CC_FAT intracellular organelle lumen(17) 18.9% 7.9E-3  GOTERM_CC_FAT organelle lumen(17) 18.9% 9.7E-3 3.4E-1  GOTERM_CC_FAT membrane-enclosed lumen(17) 18.9% 1.2E-2  GOTERM_CC_FAT endoplasmic reticulum(10) 11.1% 3.7E-2 |
| SP_PIR_KEYWORDS  P<0.05 | Sublist Category Term (Count) % P-Value  SP_PIR_KEYWORDS acetylation(27) 30.0% 1.0E-4  SP_PIR_KEYWORDS mrna splicing(6) 6.7% 2.9E-3  SP_PIR_KEYWORDS repressor(8) 8.9% 4.0E-3  SP_PIR_KEYWORDS mrna processing(6) 6.7% 7.2E-3  SP_PIR_KEYWORDS transcription regulation(17) 18.9% 2.3E-2  SP_PIR_KEYWORDS phosphoprotein(44) 48.9% 2.7E-2  SP_PIR_KEYWORDS Transcription(17) 18.9% 2.7E-2  SP_PIR_KEYWORDS nucleus(29) 32.2% 2.8E-2  SP_PIR_KEYWORDS translation regulation(3) 3.3% 3.9E-2  SP_PIR_KEYWORDS wd repeat(5) 5.6% 3.9E-2  SP_PIR_KEYWORDS endoplasmic reticulum(8) 8.9% 4.7E-2 |
| UP_SEQ_FEATURE  P<0.05 | UP_SEQ_FEATURE repeat:WD 3(5) 5.6% 3.5E-2  UP_SEQ_FEATURE repeat:WD 1(5) 5.6% 3.9E-2  UP_SEQ_FEATURE repeat:WD 2(5) 5.6% 3.9E-2  UP_SEQ_FEATURE mutagenesis site(16) 17.8% 5.0E-2 |

**(2) Genes switched to longer 3’UTR**

| GO category | Genes switched to longer 3’UTR (105) |
| --- | --- |
| GOTERM_BP_FAT  p<0.05 | Sublist Category Term (Count) % P-Value  GOTERM_BP_FAT apoptosis(12)11.4% 8.8E-4  GOTERM_BP_FAT programmed cell death(12) 11.4% 1.0E-3  GOTERM_BP_FAT protein modification by small protein conjugation or removal(6) 5.7% 2.7E-3  GOTERM_BP_FAT cell death(12) 11.4% 3.6E-3  GOTERM_BP_FAT death(12) 11.4% 3.8E-3  GOTERM_BP_FAT protein ubiquitination during ubiquitin-dependent protein catabolic process(3) 2.9% 4.5E-3  GOTERM_BP_FAT protein ubiquitination(5) 4.8% 5.6E-3  GOTERM_BP_FAT response to axon injury(3) 2.9% 6.3E-3  GOTERM_BP_FAT protein modification by small protein conjugation(5) 4.8% 8.1E-3  GOTERM_BP_FAT mitochondrion organization(5) 4.8% 9.4E-3  GOTERM_BP_FAT protein polyubiquitination(3) 2.9% 1.0E-2  GOTERM_BP_FAT ubiquitin-dependent protein catabolic process(6) 5.7% 1.5E-2  GOTERM_BP_FAT tetrahydrobiopterin biosynthetic process(2) 1.9% 3.5E-2  GOTERM_BP_FAT removal of superoxide radicals(2) 1.9% 4.1E-2  GOTERM_BP_FAT hydrogen peroxide biosynthetic process(2) 1.9% 4.1E-2  GOTERM_BP_FAT tetrahydrobiopterin metabolic process(2) 1.9% 4.1E-2  GOTERM_BP_FAT positive regulation of apoptosis(7) 6.7% 4.4E-2  GOTERM_BP_FAT positive regulation of programmed cell death(7) 6.7% 4.5E-2  GOTERM_BP_FAT positive regulation of cell death(7) 6.7% 4.6E-2  GOTERM_BP_FAT translation(6) 5.7% 4.8E-2 |
| GOTERM_CC_FAT p<0.05 | Sublist Category Term Count % P-Value  GOTERM_CC_FAT mitochondrion(13) 12.4% 3.4E-2 |
| GOTERM_MF_FAT  p<0.05 | Sublist Category Term Count % P-Value  GOTERM_MF_FAT C-acyltransferase activity(3) 2.9% 4.4E-3  GOTERM_MF_FAT oxidoreductase activity, acting on superoxide radicals as acceptor(2) 1.9% 1.9E-2  GOTERM_MF_FAT superoxide dismutase activity(2) 1.9% 1.9E-2  GOTERM_MF_FAT acetyl-CoA C-acetyltransferase activity(2) 1.9% 1.9E-2  GOTERM_MF_FAT C-acetyltransferase activity(2) 1.9% 2.5E-2  GOTERM_MF_FAT hydro-lyase activity(3) 2.9% 3.1E-2  GOTERM_MF_FAT ubiquitin-ubiquitin ligase activity(2) 1.9% 3.1E-2  GOTERM_MF_FAT acid-amino acid ligase activity(5) 4.8% 3.7E-2 |
| SP_PIR_KEYWORDS  p<0.05 | Sublist Category Term Count % P-Value  SP_PIR_KEYWORDS acetylation(27) 25.7% 1.4E-3  SP_PIR_KEYWORDS lyase(5) 4.8% 3.9E-3  SP_PIR_KEYWORDS endoplasmic reticulum(11) 10.5% 5.2E-3  SP_PIR_KEYWORDS ubl conjugation(9) 8.6% 1.4E-2  SP_PIR_KEYWORDS tetrahydrobiopterin biosynthesis(2) 1.9% 2.7E-2  SP_PIR_KEYWORDS rna-binding(8) 7.6% 2.7E-2  SP_PIR_KEYWORDS mitochondrion(10) 9.5% 3.6E-2  SP_PIR_KEYWORDS sh3-binding(3) 2.9% 4.3E-2  SP_PIR_KEYWORDS transit peptide(7) 6.7% 4.4E-2  SP_PIR_KEYWORDS protein transport(7) 6.7% 4.8E-2  SP_PIR_KEYWORDS lipid metabolism(4) 3.8% 4.9E-2 |
| UP_SEQ_FEATURE  p<0.05 | Sublist Category Term RT Genes Count % P-Value  UP_SEQ_FEATURE mutagenesis site(20) 19.0% 1.4E-2  UP_SEQ_FEATURE domain:U-box(2) 1.9% 3.7E-2  UP_SEQ_FEATURE transit peptide:Mitochondrion(7) 6.7% 4.2E-2 |
