# Supplementary material for: A Global Analysis of Tandem 3′UTRs in Eosinophilic Chronic Rhinosinusitis with Nasal Polyps
Source: PLoS One. 2012 Nov 19;7(11):e48997. doi: 10.1371/journal.pone.0048997 (PMC3501494; doi:10.1371/journal.pone.0048997)
Supplement: Table S3 — The enrichment of Gene Ontology Biological Process terms among differentially expressed genes (greater than 3-fold difference) between nasal polyp tissue and control mucosa tissue. The numbers in and after the parentheses indicate the number of genes and the percentage for a particular category, respectively. (DOCX) [file pone.0048997.s005.docx]

**Table S3. Enrichment of Biological Process terms of Gene Ontology in differentially-expressed genes (FDR=0.01, greater than 3-fold difference) between nasal polyp tissue and control mucosa tissue.** Numbers in parentheses and ones after it indicate number of genes for a particular category.

**(1) Genes upregulated in nasal polyp tissue**

| GO category | Genes upregulated in nasal polyp tissue (213) |
| --- | --- |
| GOTERM_BP_FAT FDR<0.05 | Sublist Category Term Count % P-Value Benjamini  GOTERM_BP_FAT leukocyte activation(13) 6.4% 1.9E-5 2.6E-2  GOTERM_BP_FAT cell activation(14) 6.9% 2.3E-5 1.5E-2  GOTERM_BP_FAT lymphocyte activation(11) 5.4% 8.8E-5 3.9E-2 |
| GOTERM_BP_FAT p<0.05 | Sublist Category Term Count % P-Value  GOTERM_BP_FAT defense response(19) 9.4% 2.0E-4  GOTERM_BP_FAT T cell activation(8) 3.9% 5.7E-4  GOTERM_BP_FAT T cell proliferation(4) 2.0% 3.4E-3  GOTERM_BP_FAT lymphocyte apoptosis(3) 1.5% 5.4E-3  GOTERM_BP_FAT inflammatory response(10) 4.9% 1.2E-2  GOTERM_BP_FAT lymphocyte proliferation(4) 2.0% 1.2E-2  GOTERM_BP_FAT mononuclear cell proliferation(4) 2.0% 1.3E-2  GOTERM_BP_FAT leukocyte proliferation(4) 2.0% 1.3E-2  GOTERM_BP_FAT chemical homeostasis(13) 6.4% 1.3E-2  GOTERM_BP_FAT cellular di-, tri-valent inorganic cation homeostasis(8) 3.9% 1.5E-2  GOTERM_BP_FAT cation homeostasis(9) 4.4% 1.6E-2  GOTERM_BP_FAT ion homeostasis(11) 5.4% 1.8E-2  GOTERM_BP_FAT di-, tri-valent inorganic cation homeostasis(8) 3.9% 1.9E-2  GOTERM_BP_FAT innate immune response-activating signal transduction(3) 1.5% 1.9E-2  GOTERM_BP_FAT activation of innate immune response(3) 1.5% 1.9E-2  GOTERM_BP_FAT immune response-activating signal transduction(4) 2.0% 2.1E-2  GOTERM_BP_FAT defense response to virus(3) 1.5% 2.1E-2  GOTERM_BP_FAT cofactor biosynthetic process(5) 2.5% 2.4E-2  GOTERM_BP_FAT immune response(15) 7.4% 2.5E-2  GOTERM_BP_FAT immune response-regulating signal transduction(4) 2.0% 2.5E-2  GOTERM_BP_FAT cellular cation homeostasis(8) 3.9% 2.5E-2  GOTERM_BP_FAT cellular ion homeostasis(10) 4.9% 2.6E-2  GOTERM_BP_FAT ribonucleoside monophosphate biosynthetic process(3) 1.5% 2.8E-2  GOTERM_BP_FAT cellular chemical homeostasis(10) 4.9% 2.9E-2  GOTERM_BP_FAT ribonucleoside monophosphate metabolic process(3) 1.5% 3.2E-2  GOTERM_BP_FAT response to virus(5) 2.5% 3.5E-2  GOTERM_BP_FAT lymphocyte homeostasis(3 1.5 4.0E-2 |
| GOTERM_CC_FAT p<0.05 | Sublist Category Term Count % P-Value  GOTERM_CC_FAT basolateral plasma membrane(7) 3.4 2.1E-2 |
|  | Sublist Category Term RT Genes Count % P-Value  GOTERM_MF_FAT transcription factor activity(19) 9.4% 2.7E-2  GOTERM_MF_FAT purine nucleoside binding(27) 13.3% 3.6E-2  GOTERM_MF_FAT nucleoside binding(27) 13.3% 3.9E-2 9.9E-1  GOTERM_MF_FAT protein dimerization activity(12) 5.9% 4.4E-2  GOTERM_MF_FAT adenyl ribonucleotide binding(25) 12.3% 4.9E-2 |
| Pathway  p<0.05 | Sublist Category Term Count % P-Value  BBID chemokine_receptor-ligand(2) 1.0% 4.4E-2 |
| SP_PIR_KEYWORDS  p<0.05 | Sublist Category Term Count % P-Value  SP_PIR_KEYWORDS nucleus(59) 29.1% 1.4E-2  SP_PIR_KEYWORDS autophosphorylation(4) 2.0% 1.4E-2  SP_PIR_KEYWORDS phosphotransferaseRT(7) 3.4% 1.8E-2  SP_PIR_KEYWORDS alternative splicing(92) 45.3% 2.9E-2  SP_PIR_KEYWORDS transcription regulation(30) 14.8% 4.5E-2  SP_PIR_KEYWORDS inflammatory response(4) 2.0% 4.6E-2  SP_PIR_KEYWORDS dna-binding(28) 13.8% 4.7E-2  SP_PIR_KEYWORDS phosphoprotein(88) 43.3% 4.8E-2 |
| UP_SEQ_FEATURE  p<0.05 | Sublist Category Term Count % P-Value  UP_SEQ_FEATURE domain:PDZ2(4)2.0% 8.3E-3  UP_SEQ_FEATURE domain:PDZ1(4)2.0% 8.3E-3  UP_SEQ_FEATURE domain:HelicaseC-terminal(5) 2.5% 2.4E-2  UP_SEQ_FEATURE domain:HelicaseATP-binding(5) 2.5 2.8E-2  UP_SEQ_FEATURE domain:PDZ3(3) 1.5% 3.4E-2  UP_SEQ_FEATURE sequencevariant(138)68.0% 4.2E-2  UP_SEQ_FEATURE splicevariant(91) 44.8% 4.3E-2  UP_SEQ_FEATURE nucleotidephosphate-bindingregion:ATP(17)8.4% 4.4E-2  UP_SEQ_FEATURE sho(sequencemotif:DEADbox(3)1.5% 4.6E-2 |

**(2) Genes downregulated in nasal polyp tissue**

| GO category | Genes downregulated in nasal polyp tissue (414) |
| --- | --- |
| GOTERM_BP_FAT p<0.05 | Sublist Category Term Count % P-Value  GOTERM_BP_FAT regulation of microtubule cytoskeleton organization(5) 1.3% 9.3E-3  GOTERM_BP_FAT cell projection morphogenesis(12) 3.1% 9.9E-3  GOTERM_BP_FAT coenzyme biosynthetic process(6) 1.5% 1.2E-2  GOTERM_BP_FAT cell part morphogenesis(12) 3.1% 1.3E-2  GOTERM_BP_FAT RNA modification(5) 1.3% 1.5E-2  GOTERM_BP_FAT regulation of microtubule-based process(5) 1.3% 1.6E-2  GOTERM_BP_FAT cell death(24) 6.1% 1.6E-2  GOTERM_BP_FAT apoptosis(21) 5.3% 1.7E-2  GOTERM_BP_FAT death(24) 6.1% 1.7E-2  GOTERM_BP_FAT vitamin biosynthetic process(4) 1.0% 1.7E-2  GOTERM_BP_FAT programmed cell death(21) 5.3% 1.9E-2  GOTERM_BP_FAT membrane organization(15) 3.8% 2.0E-2  GOTERM_BP_FAT regulation of microtubule polymerization or depolymerization(4) 1.0% 2.3E-2  GOTERM_BP_FAT cell morphogenesis(14) 3.6% 2.5E-2  GOTERM_BP_FAT positive regulation of microtubule polymerization or depolymerization(3) 0.8% 2.6E-2  GOTERM_BP_FAT neuron projection morphogenesis(10) 2.5% 2.6E-2  GOTERM_BP_FAT negative regulation of protein kinase activity(6) 1.5% 2.9E-2  GOTERM_BP_FAT membrane invagination(10) 2.5% 3.2E-2  GOTERM_BP_FAT endocytosis(10) 2.5% 3.2E-2  GOTERM_BP_FAT coenzyme metabolic process(8) 2.0% 3.3E-2  GOTERM_BP_FAT negative regulation of kinase activity(6) 1.5% 3.3E-2  GOTERM_BP_FAT ncRNA processing(9) 2.3% 3.3E-2  GOTERM_BP_FAT negative regulation of microtubule depolymerization(3) 0.8% 3.9E-2  GOTERM_BP_FAT regulation of microtubule depolymerization(3) 0.8% 3.9E-2  GOTERM_BP_FAT ncRNA metabolic process(10) 2.5% 4.0E-2  GOTERM_BP_FAT skeletal muscle organ development(5) 1.3% 4.2E-2  GOTERM_BP_FAT skeletal muscle tissue development(5) 1.3% 4.2E-2  GOTERM_BP_FAT regulation of vesicle-mediated transport(6) 1.5% 4.2E-2  GOTERM_BP_FAT negative regulation of transferase activity(6) 1.5% 4.2E-2  GOTERM_BP_FAT cofactor biosynthetic process(6) 1.5% 4.4E-2  GOTERM_BP_FAT negative regulation of microtubule polymerization or depolymerization(3) 0.8% 4.4E-2  GOTERM_BP_FAT negative regulation of protein complex disassembly(4) 1.0% 4.7E-2 |
| GOTERM_CC_FAT p<0.05 | Sublist Category Term Count % P-Value  GOTERM_CC_FAT mitochondrion(32) 8.1% 8.3E-3  GOTERM_CC_FAT cytoplasmic vesicle(19) 4.8% 4.6E-2  GOTERM_CC_FAT endomembrane system(22) 5.6% 4.8E-2 |
| GOTERM_MF_FAT p<0.05 | Sublist Category Term Count % P-Value  GOTERM_MF_FAT RNA methyltransferase activity(4) 1.0% 8.1E-3  GOTERM_MF_FAT ubiquitin-protein ligase activity(8) 2.0% 2.3E-2  GOTERM_MF_FAT zinc ion binding(57) 14.5% 3.1E-2  GOTERM_MF_FAT ligase activity, forming carbon-nitrogen bonds(10) 2.5% 3.4E-2  GOTERM_MF_FAT small conjugating protein ligase activity(8) 2.0% 4.0E-2 |
| UP_SEQ_FEATURE p<0.05 | Sublist Category Term Count % P-Value  UP_SEQ_FEATURE splice variant(181) 46.1% 0.00051  UP_SEQ_FEATURE zinc finger region:C2H2-type 12(14) 3.6% 0.0017  UP_SEQ_FEATURE zinc finger region:C2H2-type 13(12) 3.1% 0.0024  UP_SEQ_FEATURE domain: Sema(5) 1.3 0.0032  UP_SEQ_FEATURE zinc finger region:C2H2-type 11(14) 3.6% 0.0061  UP_SEQ_FEATURE zinc finger region:C2H2-type 17(7) 1.8% 0.0062  UP_SEQ_FEATURE domain:KRAB(15) 3.8% 0.007  UP_SEQ_FEATURE zinc finger region:C2H2-type 4(21) 5.3% 0.012  UP_SEQ_FEATURE zinc finger region:C2H2-type 5(20) 5.1% 0.012  UP_SEQ_FEATURE zinc finger region:C2H2-type 16(7) 1.8% 0.017  UP_SEQ_FEATURE zinc finger region:C2H2-type 10(14) 3.6% 0.017  UP_SEQ_FEATURE zinc finger region:C2H2-type 6(18) 4.6% 0.019  UP_SEQ_FEATURE zinc finger region:C2H2-type 9(15) 3.8% 0.019  UP_SEQ_FEATURE zinc finger region:C2H2-type 7(17) 4.3% 0.019  UP_SEQ_FEATURE zinc finger region:C2H2-type 2(21) 5.3% 0.02  UP_SEQ_FEATURE zinc finger region:C2H2-type 8(16) 4.1% 0.02  UP_SEQ_FEATURE transit peptide: Mitochondrion(17) 4.3% 0.025  UP_SEQ_FEATURE zinc finger region: RING-type(10) 2.5% 0.025  UP_SEQ_FEATURE compositionally biased region: Pro-rich(29) 7.4% 0.028  UP_SEQ_FEATURE zinc finger region:C2H2-type 1; degenerate(7) 1.8% 0.035  UP_SEQ_FEATURE zinc finger region:C2H2-type 14(8) 2% 0.038  UP_SEQ_FEATURE zinc finger region:C2H2-type 18(5) 1.3% 0.042  UP_SEQ_FEATURE zinc finger region:C2H2-type 1(18) 4.6% 0.044 |
| SP_PIR_KEYWORDS p<0.05 | Sublist Category Term Count % P-Value  SP_PIR_KEYWORDS alternative splicing(178) 45.3% 0.0013  SP_PIR_KEYWORDS coiled coil(60) 15.3% 0.0014  SP_PIR_KEYWORDS ligase(14) 3.6% 0.0079  SP_PIR_KEYWORDS zinc-finger(48) 12.2% 0.014  SP_PIR_KEYWORDS transit peptide(17) 4.3% 0.028  SP_PIR_KEYWORDS phosphoprotein(162) 41.2% 0.035  SP_PIR_KEYWORDS leucine zipper(4) 1% 0.039  SP_PIR_KEYWORDS Apoptosis(14) 3.6% 0.04 |
