# Supplementary material for: A Global Analysis of Tandem 3′UTRs in Eosinophilic Chronic Rhinosinusitis with Nasal Polyps
Source: PLoS One. 2012 Nov 19;7(11):e48997. doi: 10.1371/journal.pone.0048997 (PMC3501494; doi:10.1371/journal.pone.0048997)
Supplement: Table S4 — Validation of 3′UTR switching in nasal polyp tissue compared with control tissue using RT-PCR. ER: Expression ratios of the shortened region to the lengthened region. P: polyp tissue; C: control tissue. Pearson r: A larger positive/negative value indicates that longer/shorter tandem UTRs are prone to be used in the nasal polyp. (DOCX) [file pone.0048997.s006.docx]

**Table S4. Validation of 3’UTR switching in nasal polyp compared with control tissue using RT-PCR.**

|  | SAPAS | | | qRT-PCR | | | |
| --- | --- | --- | --- | --- | --- | --- | --- |
| UCSC ID | 3’UTR | Pearson_r**^ξ^** | FDR | 3’UTR | P-ER* | C-ER* | P- value |
| uc002sqc.2 | lengthen | 0.55641 | 2.21E-28 | lengthen | 23.2456 | 32.4118 | 0.04769 |
| uc002dfr.2 | lengthen | 0.97 | 6.89E-22 | lengthen | 9.17411 | 10.7675 | 0.0201 |
| uc003xzf.2 | lengthen | 0.56347 | 3.52E-17 | lengthen | 2.72580 | 3.31161 | 0.0366 |
| uc003ccg.2 | shorten | -0.6262921 | 6.90E-30 | shorten | 22.0893 | 20.1354 | 0.04603 |
| uc010bkb.1 | shorten | -0.2660729 | 2.89E-06 | failed |  |  |  |

*ER: Expression ratios of the shortened region to the lengthened region. P: Polyp tissue; C: control tissue.

**^ξ^**Pearson r: A larger positive/negative value indicates that longer/shorter tandem UTRs are prone to be used in the nasal polyp.
