# Supplementary material for: A Global Analysis of Tandem 3′UTRs in Eosinophilic Chronic Rhinosinusitis with Nasal Polyps
Source: PLoS One. 2012 Nov 19;7(11):e48997. doi: 10.1371/journal.pone.0048997 (PMC3501494; doi:10.1371/journal.pone.0048997)
Supplement: Table S5 — Validation of differentially expressed genes in nasal polyp tissue and control tissue using qRT-PCR. (DOCX) [file pone.0048997.s007.docx]

**Table S5. Validation of differentially expressed genes in nasal polyp tissue and control tissue using RT-PCR.**

| UCSC ID | Fold difference measured by sequencing (polyp/control) | Fold difference measured by real-time RT-PCR (polyp/control) |
| --- | --- | --- |
| uc001ehb.2([VTCN1](http://www.genecards.org/cgi-bin/carddisp.pl?gene=VTCN1)) | 0.28859 | 0.50463 |
| uc010taa.1([Diablo](http://www.genecards.org/cgi-bin/carddisp.pl?gene=Diablo)) | 0.13878 | 0.31280 |
| uc001wso.2([srp54](http://www.genecards.org/cgi-bin/carddisp.pl?gene=srp54)) | 15.1200 | 2.51984 |
| uc003aij.1([PES1](http://www.genecards.org/cgi-bin/carddisp.pl?gene=PES1)) | 12.77143 | 3.58838 |
| uc002jbd.2([TACO1](http://www.genecards.org/cgi-bin/carddisp.pl?gene=TACO1),) | 0.16667 | 0.616996 |
| uc003tmu.2([TBRG4](http://www.genecards.org/cgi-bin/carddisp.pl?gene=TBRG4)) | 0.14815 | 0.511687 |
| uc003olv.3([BRPF3](http://www.genecards.org/cgi-bin/carddisp.pl?gene=BRPF3)) | 10.36364 | 1.17555 |
| uc003vvm.2([Jhdm1d](http://www.genecards.org/cgi-bin/carddisp.pl?gene=Jhdm1d)) | 7.875 | 1.030492 |
| uc011jzi.1([skap2](http://www.genecards.org/cgi-bin/carddisp.pl?gene=skap2)) | 15.2 | 1.119872 |
| uc001hjl.1([BATF3](http://www.genecards.org/cgi-bin/carddisp.pl?gene=BATF3)) | 10 | 0.799221 |
