# Supplementary material for: A Global Analysis of Tandem 3′UTRs in Eosinophilic Chronic Rhinosinusitis with Nasal Polyps
Source: PLoS One. 2012 Nov 19;7(11):e48997. doi: 10.1371/journal.pone.0048997 (PMC3501494; doi:10.1371/journal.pone.0048997)
Supplement: Table S6 — Primers used for the SAPAS library construction. (DOCX) [file pone.0048997.s008.docx]

**Table S6. Primers used in SAPAS library construction.**

| **Primer ID** | Sequences |
| --- | --- |
| **PrimerB-poly(A)VN** | GCCTTGCCAGCCCGCTCAG(T)20VN |
| **PrimerB-poly(A)20-2cVN** | GCCTTGCCAGCCCGCTCAGTTTTTTcTTTTTTcTTTTTTVN |
| **PrimerA-GGG** | GCCTCCCTCGCGCCATCAGrGrGr |
| **PrimerA-GGG_with_A** | GCCTCCCTCGCGCCATCAGAGG |
| **Illumina_Primer1_poly(A)20VN** | ACACTCTTTCCCTACACGACGCTCTTCCGATC(T)20VN |
| **Illumina_Primer1_Poly(A)20_2cVN** | AATGATACGGCGACCACCGAGATCTACACTCTTTCCCTACACGACGCTCTTCCGATCTTTTTTcTTTTTTcTTTTTTVN |
