# Supplementary material for: A Global Analysis of Tandem 3′UTRs in Eosinophilic Chronic Rhinosinusitis with Nasal Polyps
Source: PLoS One. 2012 Nov 19;7(11):e48997. doi: 10.1371/journal.pone.0048997 (PMC3501494; doi:10.1371/journal.pone.0048997)
Supplement: Table S7 — PCR primers used in qRT-PCR. (DOCX) [file pone.0048997.s009.docx]

**Table S7. PCR primers used in qRT-PCR.**

| qRT-PCR primers for APA switching genes | |
| --- | --- |
| uc002sqc.2([c2orf68](http://bioinfo.weizmann.ac.il/cards-bin/carddisp?c2orf68))-short-F | 5'TTCTCACCGAGGTCATAT3' |
| uc002sqc.2([c2orf68](http://bioinfo.weizmann.ac.il/cards-bin/carddisp?c2orf68))-short-R | 5'GAGGGAAAGGAACAAAGA3' |
| uc002sqc.2([c2orf68](http://bioinfo.weizmann.ac.il/cards-bin/carddisp?c2orf68))-long-F | 5'ATGGTGAGTATCCAATCT3' |
| uc002sqc.2 ([c2orf68](http://bioinfo.weizmann.ac.il/cards-bin/carddisp?c2orf68))-long-R | 5'AATCTGTACTTATTCCTCTT3' |
| uc002dfr.2([coq7](http://bioinfo.weizmann.ac.il/cards-bin/carddisp?coq7))-short-F | 5'CTGATGACTTGGCAGGACTTG3' |
| uc002dfr.2([coq7](http://bioinfo.weizmann.ac.il/cards-bin/carddisp?coq7))-short-R | 5'GAGAGAGGTGGCAGAGGATT3' |
| uc002dfr.2([coq7](http://bioinfo.weizmann.ac.il/cards-bin/carddisp?coq7))-long-F | 5'GGGTGATAATGGGTGGGAGAG3' |
| uc002dfr.2([coq7](http://bioinfo.weizmann.ac.il/cards-bin/carddisp?coq7)))-long-R | 5'ACACTGTGGAGAGGATGAGGTA3' |
| uc003xzf.2([C8orf84](http://bioinfo.weizmann.ac.il/cards-bin/carddisp?C8orf84))-short-F | 5'TGTGTCGGTTATGGAAGG3' |
| uc003xzf.2([C8orf84](http://bioinfo.weizmann.ac.il/cards-bin/carddisp?C8orf84))-short-R | 5'GAGTTCTGGTTGAGGAGTC3' |
| uc003xzf.2([C8orf84](http://bioinfo.weizmann.ac.il/cards-bin/carddisp?C8orf84))-long-F | 5'GCCACCAGGAAAGAAGGGAAA3' |
| uc003xzf.2([C8orf84](http://bioinfo.weizmann.ac.il/cards-bin/carddisp?C8orf84))-long-R | 5'ATTGCTGTTGCGGTTTATGCTTAA3' |
| uc003ccg.2([Ube2e2](http://bioinfo.weizmann.ac.il/cards-bin/carddisp?Ube2e2))-short-F | 5' ACTGAAGTCTTTATTGGTGGGTGC 3' |
| uc003ccg.2([Ube2e2](http://bioinfo.weizmann.ac.il/cards-bin/carddisp?Ube2e2))-short-R | 5' TGCGGGAGGGACAGAAGG 3' |
| uc003ccg.2([Ube2e2](http://bioinfo.weizmann.ac.il/cards-bin/carddisp?Ube2e2))-long-F | 5' CACTGTGGCTGATAACTG 3' |
| uc003ccg.2([Ube2e2](http://bioinfo.weizmann.ac.il/cards-bin/carddisp?Ube2e2))-long-R | 5' CACTTGAACCTGTAATTGAATT 3' |
| uc010bkb.1(CSK)-short-F | 5' AGTCTATGAAGTCATGAAGAAC 3' |
| uc010bkb.1(CSK)-short-R | 5' AGTCTATGAAGTCATGAAGAAC 3' |
| uc010bkb.1(CSK)-long-F | 5' ATTCTGTGTCCCATGT 3' |
| uc010bkb.1(CSK)-long-R | 5' CACACGAAATCCAAGC3' |
| uc002ypa.2(SOD1)-short-F | 5' agacaggaaacgctggaagt 3' |
| uc002ypa.2(SOD1)-short-R | 5' gcaggataacagatgagttaaggg 3' |
| uc002ypa.2(SOD1)-long-F | 5' cctgtagtgagaaactgatttatg 3' |
| uc002ypa.2(SOD1)-long-R | 5' caggcttgaatgacaaagaaa 3' |
| uc003vej.2(BCAP29)-short-F | 5' gattcagggccaggctgtaagtct 3' |
| uc003vej.2(BCAP29)-short-R | 5' cagttgggcacggtggtaaagg 3' |
| uc003vej.2(BCAP29)-long-F | 5' ttcttcctctgtcctgctgcttga 3' |
| uc003vej.2(BCAP29)-long-R | 5' ggtgtggtgcttatggcgtcag 3' |
| uc009wty.2(DEDD)-short-F | 5' ccgcctttcctccattcca 3' |
| uc009wty.2(DEDD)-short-R | 5' cagcaacctcttctccatcca 3' |
| uc009wty.2(DEDD)-long-F | 5' tgggcaaagggcatcatag 3' |
| uc009wty.2(DEDD)-long-R | 5' cctcctggctcattcaacat 3' |
| uc011jzo.1(TAX1BP1)-short-F | 5' agaccactgaggagaccatagag 3' |
| uc011jzo.1(TAX1BP1)-short-R | 5' gataagccaaagactgaccctgat 3' |
| uc011jzo.1(TAX1BP1)-long-F | 5' ggagctgcatgtagaatga 3' |
| uc011jzo.1(TAX1BP1)-long-R | 5' actgacatctcttgttccatt 3' |
| qRT-PCR primers for differentially expressed genes | |
| uc003aij.1(PES1)-F | 5'ATGCTAATTGCTATGGTCTCATT3' |
| uc003aij.1 (PES1)-R | 5'GCAGAGTGGAACATTATTGTAAGA3' |
| uc001wso.2 ([srp54](http://bioinfo.weizmann.ac.il/cards-bin/carddisp?srp54))-F | 5'AACTAGACAGTACGGATGGT3' |
| uc001wso.2([srp54](http://bioinfo.weizmann.ac.il/cards-bin/carddisp?srp54))-R | 5'ACTGGCTCACATTCTTAGACA3' |
| uc010taa.1 (Diablo)-F | 5'CGAGAAGAGGCGGCAGAT3' |
| uc010taa.1 (Diablo)-R | 5'GACCACAGGAGGCACTCA3' |
| uc001ehb.2 (VTCN1)-F | 5'GCAAGCCAAGTTCTGTAAGAG3' |
| uc001ehb.2 (VTCN1)-R | 5'GCCTCAATTCAAGCAGTCATT3' |
| uc002jbd.2([TACO1](http://bioinfo.weizmann.ac.il/cards-bin/carddisp?TACO1))-F | 5'ACATTGAATAACCAGGCTACAT3' |
| uc002jbd.2 ([TACO1](http://bioinfo.weizmann.ac.il/cards-bin/carddisp?TACO1))-R | 5'GGCTTTACCCTCAGAGATTG3' |
| uc003tmu.2([TBRG4](http://bioinfo.weizmann.ac.il/cards-bin/carddisp?TBRG4))-F | 5'TCAACAGCCGAAGCAAGG3' |
| uc003tmu.2 ([TBRG4](http://bioinfo.weizmann.ac.il/cards-bin/carddisp?TBRG4))-R | 5'AGTTCCAGCCACTCATAGAATG3' |
| uc003olv.3([BRPF3](http://bioinfo.weizmann.ac.il/cards-bin/carddisp?BRPF3))-F | 5'GCTGTGGACTCTGTGACCTT3' |
| uc003olv.3([BRPF3](http://bioinfo.weizmann.ac.il/cards-bin/carddisp?BRPF3))-R | 5'CTGAGCCGTTTCCCGTTCTA3' |
| uc003vvm.2([Jhdm1d](http://bioinfo.weizmann.ac.il/cards-bin/carddisp?Jhdm1d))-F | 5'GCATACTGTTATCCAGGTTGAT3' |
| uc003vvm.2 ([Jhdm1d](http://bioinfo.weizmann.ac.il/cards-bin/carddisp?Jhdm1d))-R | 5'GTTCTTACACATTGGCTACGA3' |
| uc011jzi.1([skap2](http://bioinfo.weizmann.ac.il/cards-bin/carddisp?skap2))-F | 5'ATGCTAATTGCTATGGTCTCATT3' |
| uc011jzi.1([skap2](http://bioinfo.weizmann.ac.il/cards-bin/carddisp?skap2))-R | 5'GCAGAGTGGAACATTATTGTAAGA3' |
| uc001hjl.1([BATF3](http://bioinfo.weizmann.ac.il/cards-bin/carddisp?BATF3))-F | 5'GAGGATGATGACAGGAAGGT3' |
| uc001hjl.1([BATF3](http://bioinfo.weizmann.ac.il/cards-bin/carddisp?BATF3))-R | 5'TTCTTGCTCCAGGCTCTC3' |
